# Exploring Intracellular Se Speciation in Marine Phytoplankton by LC-ICP-MS/MS: from Se Enrichments to Extraction and Chromatographic Techniques

**DOI:** 10.1007/s12011-026-05069-7

**Published:** 2026-04-21

**Authors:** Pauline Béziat, Sylvain Bouchet, Julie Tolu, Uria Alcolombri, Lenny H. E. Winkel

**Affiliations:** 1 Institute of Biogeochemistry & Pollutant Dynamics, Department of Environmental System Sciences, Swiss Federal Institute of Technology (ETHZ), Universitätstrasse 16, Zurich, 8092 Switzerland; 2 Department Water Resources and Drinking Water, Swiss Federal Institute of Aquatic Science & Technology (Eawag), Überlandstrasse 133, Dübendorf, 8600 Switzerland; 3 The Alexander Silberman Institute of Life Sciences, The Hebrew University of Jerusalem, Edmon J. Safra Campus – Givat Ram, Jerusalem, 9190401 Israel; 4 Federal Institute of Metrology (METAS), Lindenweg 50, Köniz, 3003 Switzerland

## Abstract

Marine phytoplankton play a key role in biogeochemical selenium (Se) cycling. By transforming inorganic Se into organic species, many of which remain uncharacterized, they influence Se availability for other organisms and may contribute to Se volatilization. Characterizing organic Se species in phytoplankton is challenging and no standard analytical workflow has been established. Here, we used the marine phytoplankton species *Gephyrocapsa huxleyi* and *Effrenium voratum* to systematically assess how cultivation (Se concentrations in the medium), cell harvesting, extraction, and chromatographic parameters influence Se speciation measurements quantitatively (extraction yields, column recoveries) and qualitatively (species distribution), when performed by HPLC-ICP-MS/MS. We show that medium Se concentrations and choice of chromatography had the largest effect on measured Se speciation. The two phytoplankton showed marked differences in intracellular Se speciation, likely reflecting a dissimilar selenometabolism. Resolving these speciation differences was only possible with select chromatographies (e.g. anion-exchange but not mixed-mode). Both phytoplankton also showed changes to intracellular Se speciation when grown in Se-enriched media in comparison with near-natural concentrations. The selection of Se concentrations in the medium is therefore crucial for studying Se metabolites, e.g. under environmentally relevant conditions. We recommend extraction–chromatography workflows tailored to specific experimental needs in phytoplankton Se research, providing a framework for future studies to uncover further effects these organisms have on biogeochemical Se cycling.

**Graphical Abstract:**

**Figure:**
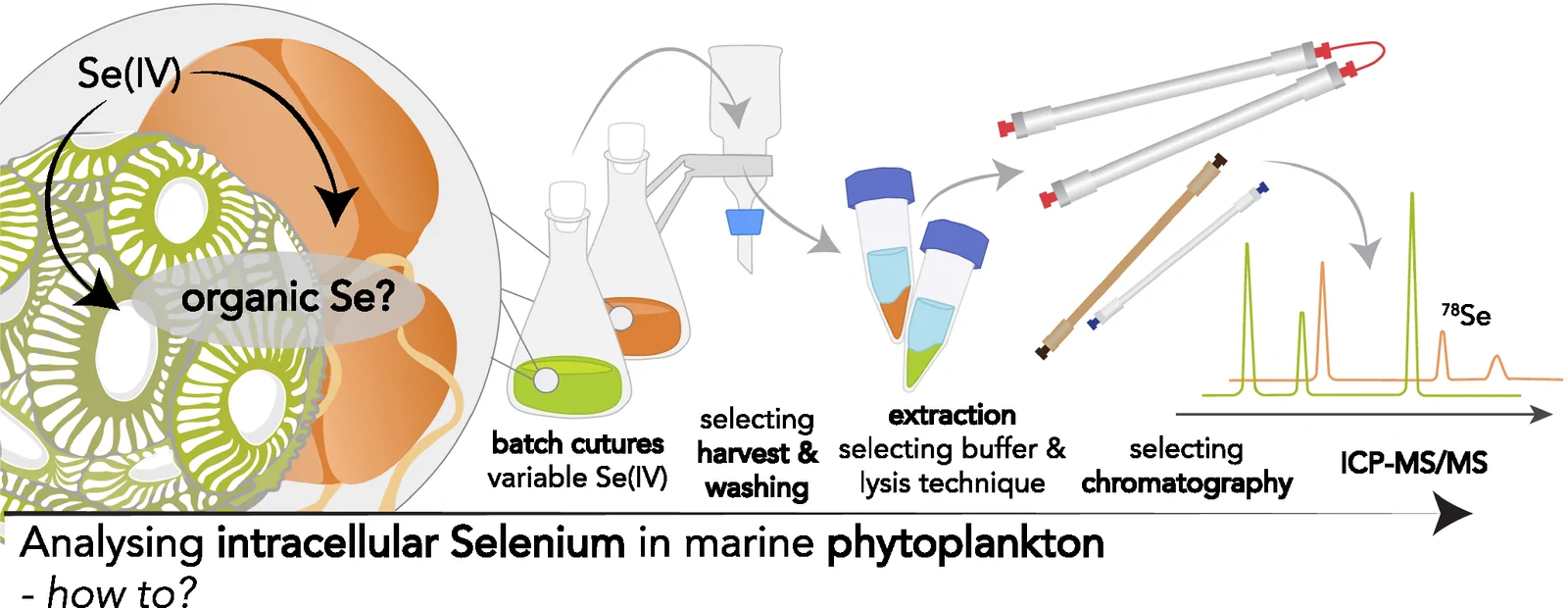

**Supplementary Information:**

The online version contains supplementary material available at https://doi.org/10.1007/s12011-026-05069-7.

## Introduction

### Why Study Se in Phytoplankton?

Marine phytoplankton are a set of diverse auto- or mixotrophic microorganisms that drive the oceanic biological pump and strongly influence global carbon cycling [1–4]. Beyond carbon, phytoplankton primary production is also at the center of other biogeochemical cycles: forming the base of the marine food web, it represents the first entry point for the bioaccumulation and biomagnification of various elements, including the trace element selenium (Se) [5–9]. Selenium is a known micronutrient for organisms across all kingdoms of life, but also acts as a toxin at elevated concentrations [10].

Phytoplankton can transform inorganic selenite (Se[IV]) present in low nM concentrations in surface (sea)waters into diverse yet largely uncharacterized organic Se compounds [11–15]. These phytoplanktonic organic Se compounds are thought to impact the wider biogeochemical Se cycle via two mechanisms: first, organic Se may be transferred to higher trophic levels in the marine food chain [7, 8, 16]. Second, it has been suggested that phytoplankton derived organic Se compounds are possible precursors for the generation of dimethyl selenide (DMSe) [17–19], a volatile Se compound that accounts for the largest natural flux of this element from seawater into the atmosphere [20].

In addition to affecting Se biogeochemistry, phytoplankton Se metabolism may influence bloom dynamics, as various species have obligate Se growth requirements [21–25] and bioavailable Se(IV) is often strongly depleted in surface seawaters, especially during phytoplankton bloom events [26–28]. There is evidence for phytoplankton assimilating both inorganic Se(IV) and organic Se species, including compounds released from other phytoplankton cells [29], which may be more bioavailable than inorganic forms [30]. However, the identities of such organic Se species and their role in the ocean remain poorly understood. Exploring the accumulation and metabolism of Se by marine phytoplankton into organic compounds will help clarify the role of these organisms in the marine Se cycle and further elucidate ecological consequences of phytoplankton – Se interactions.

### Selenometabolism in Marine Phytoplankton – Knowns and Unknowns

In biological systems, Se is generally known to be incorporated into different cellular fractions including proteins, nucleic acids and low molecular weight (LMW) metabolites [31]. In phytoplankton specifically, the majority of Se appears to accumulate in the protein fraction; in the marine coccolithophore *Gephyrocapsa huxleyi* for example, about 75% of intracellular Se was found in proteins, with the rest split between an operationally defined polysaccharides & nucleic acids fraction as well as an unknown pool of LMW metabolites [11]. While bioinformatic tools have proven fruitful in uncovering and partially characterizing phytoplankton Se-proteomes [32–35], pathways of Se incorporation into polysaccharides and LMW metabolites remain poorly understood, even though these compounds may be of particular importance from a biogeochemical perspective.

Previous studies experimentally investigating intracellular Se species in phytoplankton cultures identified the Se-amino acids selenomethionine (SeMet), selenocysteine (SeCys), or their derivatives (methylselenocysteine (MeSeCys) and oxidized selenomethionine (SeOMet)) (Table S1 for an overview of previous studies and their results) [12–15, 36–39]. It is however unclear to what extent these results are representative for intracellular Se speciation of natural marine phytoplankton, since previous studies (i) have focused almost exclusively on green lineage plastid phytoplankton, (ii) were mostly conducted using severe, artificial Se enrichment to compensate for lack of instrumental sensitivity, and (iii) used an array of sample preparation and metabolite detection techniques for which the extraction capacity and effects on Se speciation were not characterized.

The modern ocean is dominated by phytoplankton of the red plastid lineage (e.g. diatoms, dinoflagellates or coccolithophores) [40], which appear to have a relatively distinct Se-metabolism from many of those in the green plastid lineage based on a higher abundances of Se-proteins [32], and higher Se growth requirements on average [11, 22, 27, 33, 41, 42]. LMW Se speciation in red marine phytoplankton to the best of our knowledge has only been investigated in one previous study which detected 5 LMW Se-compounds in *G. huxleyi*, none of which were the aforementioned Se-amino acids SeMet or SeCys, which have been suggested to exist in e.g. *Chlorella* and remained unidentified [11].

Further, it is unclear to what extent artificial Se enrichment – often required for detection and identification of Se compounds in algae by mass spectrometry (MS), especially in the case of high resolution (HR)-MS (e.g. [38, 39]) - impacts the result of laboratory studies recording phytoplankton Se speciation. While it is well established that Se is required for, or can promote, algal growth at low concentrations (ng L^− 1^ range) [21, 23, 43], it is also understood that Se becomes toxic at higher concentrations [44–47], and can induce a variety of severe physiological effects including alterations to growth rates and cell yields [45, 46, 48–50], cell ultrastructure [51], or metabolic processes [52, 53]; precipitation of Se-rich granules (presumably in the form of elemental Se, Se(0)) has also been observed [19, 44]. These pronounced Se-dependent physiological responses raise concerns about the ecological relevance of results obtained from highly Se-enriched cultures. It remains unclear whether organic Se compounds produced under such artificial conditions (high µg to mg Se L^− 1^) are also formed at concentrations representative of the natural marine environment (pg to ng L^− 1^) [26, 54–56], or instead part of a distinct metabolic response to Se toxicity.

Finally, an additional unknown in phytoplankton Se research is the extent to which sample preparation techniques affect the results of Se speciation measurements. Previous studies have employed a range of sample preparation and analytical techniques for the extraction and detection of Se compounds, with unknown Se recoveries and impacts on Se speciation. A structured assessment of how cultivation conditions (notably Se concentrations), sample harvesting and processing and lastly chromatographic separation affect phytoplankton intracellular Se speciation measurements is lacking.

### Scope of the study

The present study aimed to evaluate the impact of experimental design on the analytical results obtained when studying Se-metabolism in marine phytoplankton. As model organisms, we used the two abundant marine red-lineage phytoplankton species *G. huxleyi*, a coccolithophorid, as well as *Effrenium voratum*, a dinoflagellate. The genomes of *G. huxleyi* and members of the *Symbiodiniaceae* (such as *E. voratum*) family have previously been searched for Se-protein encoding genes and both were found to encode Se-proteins but belonging to different families; we hypothesized that their Se-metabolism may show differences [32]. To assess the algal Se-metabolism, we employed liquid chromatography coupled to inductively coupled plasma tandem MS (LC-ICP-MS/MS). In contrast to LC-HR-MS, LC-ICP-MS/MS does not provide structural identification of individual Se compounds as it detects based on elemental composition and retention time only. While retention times can be compared to analytical standards, identification is unfeasible as hundreds of organic Se compounds are thought to exist but only a handful are commercially available. Nonetheless, LC-ICP-MS/MS is uniquely suited for explorative analyses of Se in biological matrices, as in contrast to HR-MS, the full cellular selenome (Se in proteins, sugars, LMW species etc.) can be mapped quantitatively given the superior analytical sensitivity of ICP-MS, its element-specific detection and compound independent ionization efficiency.

Here, we systematically investigated how growth conditions (Se exposure), cell harvesting, washing and extraction protocols, and chromatographic separation influence phytoplankton Se speciation measurements qualitatively and quantitatively. Quantitatively, we determined Se extraction efficiencies for different extraction protocols, and link these results to Se distributions across cellular fractions obtained from sequential extractions. We then evaluated Se speciation in these different types of extracts, assessed via four types of chromatography representing different separation mechanisms (size-exclusion, anion-exchange, cation-exchange and mixed mode chromatography). Finally, we illustrate changes in algal cellular Se distribution and speciation of LMW Se compounds when increasing Se exposure stepwise by 6 orders of magnitude, from a near natural Se(IV) concentrations of 100 ng L^− 1^ up to 100 mg L^− 1^ i.e. the Se level at which most previous studies were performed.

## Materials and Methods

### Cultivation of Phytoplankton: Growth Conditions, Growth Rate and Photosynthetic Efficiency Measurements

For experiments investigating harvesting and extraction techniques, *Effrenium voratum* (CCMP421) (formerly referred to as *Symbiodinium voratum*) and *Gephyrocapsa huxleyi* (RCC1258, non-calcifying) (formerly referred to as *Emiliania huxleyi*) were grown in autoclaved 1.2 L glass Erlenmeyer flasks equipped with cellulose stoppers and filled with 800 mL of 0.2 μm-filtered and autoclaved artificial seawater (Instant Ocean (30 PSU salinity)). The medium was amended with filter-sterilized f/2 nutrients (National Center for Marine Algae and Microbiota) and 100 µg L^− 1^ Se(IV), which is known to be the primary bioavailable form of inorganic Se for marine microalgae [57]. It is also known to be relatively resistant to photooxidation in surface seawater under environmental conditions, e.g. does not readily transform to Se(VI) [54]. Cultures were kept in a Snijders DeLUX Climate Chamber at 21 °C, 120 µmol m^− 2^ s^− 1^ photosynthetically active radiation at a 16:8 light: dark regime. Prior to experiments, cultures were maintained in a medium solution of 100 µg L^− 1^ Se(IV) for at least three transfers for acclimation.

For experiments investigating the effects of Se concentration, Instant Ocean was substituted by artificial seawater (AQUIL) (35 PSU, 450 mL per replicate in 1.2 L glass Erlenmeyer flasks) prepared according to Price et al., [58] with modifications as in Wake et al., [21]; this was chosen as Instant Ocean is a natural product (dried sea salt) that likely contains low levels of Se, and is hence not suitable for experiments necessitating precise, low Se additions. In this case, phytoplankton cultures were acclimatized to the lowest Se concentration to be used in the experiment (of 0.1 µg Se L^− 1^) for three transfers. For the experiment, cultures were amended with filter sterilized Aquil nutrients and Se(IV) at concentrations of 0.1, 1, 10, or 100 µg Se L^− 1^; we hypothesized that this range should already cover the shift from Se deficiency to toxicity for our tested algae. Nonetheless, we also included an additional treatment of 100 mg Se L^− 1^ in order to compare with previous studies that used this concentration level for metabolite detection (see Table S1). However, the growth of *E. voratum* was very poor at 100 mg Se L^− 1^, hence in the experiment of *G. huxleyi*, an additional intermediate concentration of 1 mg Se L^− 1^ was included. Cultures were maintained in a ClimeCab 1400 (Kälte 3000) at 21 °C and 100 µmol m^− 2^ s^− 1^ for 16 h per day.

The phytoplankton strains used in this study were not axenic but monoalgal, no attempt was made to quantify bacterial biomass in algae cultures. Cell growth was monitored by recording chlorophyll a fluorescence (excitation 460 nm/emission 680 nm, integration time 32 ms) using a plate reader (Tecan) or flow cytometry. Before harvesting cultures in the stationary growth phase, absolute cell counts were determined using a CytoFLEX S flow cytometer (Beckman Coulter) equipped with an air-cooled 488 nm laser and standard optical filter set-up. Samples were measured in analytical triplicates and gating was performed on an FSC vs. PC-5.5 dot plot (acquisition thresholds and gains of 3231 and 74 for FSC and 449 and 40 for PC-5.5, respectively, acquisition time: 60 s). For the Se concentration experiments, at two time points during the experimental phytoplankton growth period, a Water-PAM II (Walz) was used to record photochemical efficiencies (Fv/Fm) of phytoplankton cultures (after dark-adaptation for 30 min) in quartz cuvettes.

### Phytoplankton Biomass Harvesting

In this study, the objective was to minimize biological variability to be able to assess the variability of the extraction method used, hence all biological replicate cultures (3 to 7) were pooled and thoroughly mixed before being aliquoted for harvesting. We first tested the effect of using different cell harvesting and washing protocols, here cells were harvested by (i) filtration using an acid-washed glass filtration tower equipped with GF/C filters (Whatman) - care was taken to avoid filters falling dry - or (ii) centrifugation performed in acid-washed, conical 200 mL polypropylene Nalgene centrifugation flasks (5700 *g*, 20 min, 4 °C) in a pre-cooled Mistral 6000 centrifuge (MSE, France). Filters were washed using 50 or 100 mL of an ice-cooled washing solution containing 0.6 M NaCl (puriss, Sigma-Aldrich) and 2.38 mM NaHCO_3_ nM [59], additionally amended with 25 mM PO_4_^3−^ (Na_2_HPO_4_ and NaH_2_PO_4_ 2xH_2_O, Merck (EMSURE) and Fluka (for analysis, > 99%), respectively (pH 7.5). For centrifuged samples, phytoplankton pellets were resuspended in the same ice-cooled washing solution, transferred into 5 mL Eppendorf tubes and re-centrifuged at 3800 *g* for 10 min at 4 °C in a Sigma 3-16KL. This washing procedure was repeated up to three times to obtain the lowest carryover of Se(IV) from growth media into final samples.

### Extraction of Phytoplankton Cells for Se Speciation Measurements

We investigated different protocols for the extraction of organic intracellular Se from phytoplankton pellets, consisting out of different combinations of quenching and extraction techniques with different extraction solutions. Most of these protocols started with rapidly quenching pellets in liquid nitrogen (LN), right after removing the washing solution supernatant. Then, for the different extraction protocols, different extraction buffers or solvent (all kept on ice) were added, including (i) T = 30 mM Tris-HCl (VWR, ultrapure), pH adjusted to 7.5 using NH_3_ (Sigma, puriss); (ii) T-SDS = 30 mM Tris-HCl with 1% (w/v) sodium dodecyl sulfate (SDS) (Fluka, Ultra for mol. Bio > 99%), (iii), G = 30 mM Glycine, pH 2.5 (Fluka, > 99%); and (iv) M = Methanol (Sigma-Aldrich, LC-MS grade). After their addition, samples were vortexed and incubated on ice for at least 4 h. For some extraction protocols, additional cell lysis techniques were used:

(i) BB = Cryogenic bead beating, a mechanical cell disruption method in which cells are lysed by high-velocity collisions with ceramic beads. Cell pellets together with extraction solvents were transferred into 2 mL cryovials (Omni) and 0.6 g of ceramic beads (1.4 mm, Omni) were added. Bead beating was performed using a BeadRuptor Elite (Omni) in intervals of 10 s at 6 m s^− 1^ with 15 s breaks for a total of 36 intervals, i.e. 15 min. A continuous flow of liquid nitrogen maintained the temperature in the bead beating chamber at −4 °C.
(ii) Sonic = Sonication, using a tip sonicator (Sonics). Samples were sonicated on ice for 3 min at 30% intensity with pulses of 0.5 s followed by 2 s pause.
(iii) Micro = Microwaving, using a Discover SP-D Clinical microwave (CEM). Samples were transferred into 15 m glass vials with PTFE inlets and PTFE stir bar. They were treated in the microwave for 10 min at 65 °C followed by a 2 min temperature ramping phase.
(iv) Dris = Enzymatic extraction using driselase (from Basidiomycetes, Sigma-Aldrich). An 8% (w/v) driselase solution was prepared in 30mM Tris-HCl and added into samples to reach a final driselase concentration of 4% (w/v). Samples were incubated for one hour at room temperature with continuous stirring using PTFE stir bars.

Following the extraction, samples were recentrifuged at 3800 g for 15 min at 4 °C and the supernatant was filtered using a 0.22 μm syringe cellulase acetate filter. In the extraction protocol that used MeOH as a solvent, the extract was evaporated under N_2_ and the residual was taken up in 30 mM Tris-HCl in order to avoid injecting high volumes of MeOH into the HPLC-ICP-MS/MS. This was necessary, since MeOH strongly disturbs the elution of several Se species on the mixed-mode column used in this work (see Fig. S1) and further requires the addition of oxygen to avoid plasma extinction, largely lowering the instrument sensitivity.

All samples were stored in the fridge until analysis the same day, or frozen at −20 °C in between analyses.

### Se Distribution Across Cellular Fractions and Extraction Efficiencies

The fractionation procedure of Obata et al., [11] was followed with minor modifications for the assessment of Se distribution across intracellular fractions, i.e. proteins, lipids, metabolites (LMW compounds) as well as polysaccharides & nucleic acids. Briefly, centrifuged and washed cell pellets were flash frozen in liquid nitrogen, resuspended in methanol: chloroform (2:1) mix resulting in the formation of a white precipitate (dependent on the amount of biomass to be extracted, min. 3 mL) and centrifuged at 3800 g for 15 min. The supernatant was decanted and amended with a 0.88% KCl solution (volume used = 0.6 x volume of methanol: chloroform used), inducing phase separation between the aqueous metabolite and the organic lipid fraction. The pellet was boiled in 5% trichloroacetic acid (volume used = 0.33 x volume of methanol: chloroform used) for 30 min and again centrifuged to yield the proteins (precipitate) and polysaccharides & nucleic acids (supernatant). The lipid containing chloroform fraction was allowed to evaporate.

Intracellular fractions, unextracted cell pellets or cell extracts were acid-digested in PTFE tubes using an MLS Milestone microwave digestor (Ultraclave high performance microwave reactor, MLS GmbH, Leutkirch, Germany) prior to total Se measurements by ICP-MS/MS (see supplementary note S2 for details of the program). In case of solid samples or lipid residues, 0.5 mL HNO_3_ (suprapure, 65%, Sigma-Aldrich) were added to sample vials, thoroughly vortexed and transferred into a PTFE microwave vessel, the same process was repeated with another 0.5 mL HNO_3_ and 2 × 0.5 mL H_2_O_2_ (suprapure, 30%, Sigma-Aldrich). In case of liquid samples, aliquots were directly transferred into microwave tubes; HNO_3_ and H_2_O_2_ were then added. 60 mg IAEA-392 (*Scenedesmus obliquus*) was used as a standard reference material. Prior to measurements, all samples were diluted to a final HNO_3_ concentration of 6.5% (10% v/v) using ultrapure water.

### Total Se Measurement by ICP-MS/MS

Se quantification was accomplished using an ICP-MS/MS (Agilent 8800 or Agilent 8900) equipped with a conical nebulizer (Meinhard), a double-pass spray Scott chamber (Agilent) cooled at 2 °C and Pt skimmer and sampler cones. The operating conditions are provided in Table S2. All ICP-MS/MS parameters were optimized daily using a tuning solution containing 5 µg L^− 1^ Li, Y, Co and Te whereas a solution containing 50 µg L^− 1^ Y and 1 mg L^− 1^ Sc was used for drift correction during the analysis. Every 10 and 13 samples, respectively, performance was assessed by quantifying the recovery of a QC (5 µg L^− 1^ Se standard) and standard reference material (IAEA-392). Further information regarding the use of this standard reference material, recoveries and choices of internal standard (ISTD) can be found in supplementary note S2. For Se quantification, we monitored all isotopes between 76 and 82 in H_2_ (on mass) and O_2_ (+ 16 mass shift) mode, in H_2_ mode, intensities of ^77^Se & ^78^Se and ^80^Se & ^82^Se were corrected for ^1^H^76^Se & ^1^H^77^Se and ^1^H^79^Br & ^1^H^81^Br interferences, respectively, using hydridation factors (fSe and fBr) determined in the same batch on every analysis day according to Tolu et al., [60]. We aimed to include the maximum number of isotopes that showed reasonable agreement, for most samples 77, 80 and 82 (H_2_ mode) provided robust results, yielding an average agreement between considered isotopes across all samples and batches of 7.5 ± 3.7%. We however note that for such high matrix sample, manual data curation is essential, selected samples require an exclusion of isotope 82 and allowed for the inclusion of isotope 76 or vice versa (see SI Note 1 for further information).

### HPLC-ICP-MS/MS Analysis

Analysis of Se speciation in phytoplankton extracts was performed by coupling HPLC (Agilent 1260 Infinity) to either an Agilent 8800 or Agilent 8900 (for the size-exclusion chromatography (SEC) analysis) run in H_2_ mode. An ISTD solution containing 1 mg L^− 1^ Sc and 50 µg L^− 1^ Y was infused post-column to monitor sensitivity drift during the analysis. Se calibration curves (0.1–50 µg L^− 1^) were prepared from stock (5000 µg L^− 1^) solutions kept at 4 °C: selenomethionine (SeMet, Sigma-Aldrich), selenocystine (SeCys_2_, Sigma-Aldrich), selenite (Se(IV), Spectracer), selenate (Se(VI), Spectracer), methylselenocysteine (MeSeCys, Sigma-Aldrich), selenoethionine (SeEt, Toronto Research Chemicals), methane seleninic acid (MSeA, Sigma-Aldrich), benzeneseleninic acid (PhSeOOH, Sigma-Aldrich) and 2-(methylseleno)cinnamic acid (SeCinna, Sigma-Aldrich). Trimethylselenonium (TMSe) was kindly provided by Katrin Löschner (National Food Institute, Denmark), and dimethylseleniopropionate (DMSeP) and methylselenomethionine (MeSeMet) were synthesized in-house according to Fan et al., 1998 and characterized using LC-Orbitrap and nuclear magnetic resonance spectroscopy (NMR) [61]. The standards were selected based on commercial availability to cover a broad range of compounds within the limited set of available organo-Se standards. Additionally, hypothesis-driven targets were included; for example, SeEt and DMSeP were previously reported in *Chlorella* extracts [39]. Columns used for the HPLC analysis and their respective chromatographic conditions are listed in Table 1, these columns and gradients were chosen based on their widespread usage in the field, as the objective was to test existing Se chromatographic methods for their utility in phytoplankton extracts. The cation-exchange column with pyridine formate gradient was previously utilized for Se speciation in yeast and *Chlorophyceae* [39], the mixed-mode column is widely used for Se speciation in general [62, 63], and was e.g. previously employed with a similar mobile phase for organic Se speciation in lake water or soils [63, 64]. Also, anion-exchange chromatography has been widely used for Se speciation in many different matrices; in this case we base our chromatography on the methods by Darrouzès et al., 2005, further refined by Breuninger et al., 2024 [65–67]. For SEC, the Superdex peptide columns were chosen for their successful application in the study of Se speciation in various biological matrices such as cereal crops [68] or internal tissue of seabirds [69]. Protein and polystyrene sulfonic acid size standards for the SEC analysis were purchased from Polymer Standards Service (PSS, now Agilent).

**Table 1:** HPLC columns employed in this study for the analysis of Se speciation in marine microalgae. Please refer to Table S2 for the anion-exchange and cation-exchange gradient settings

Type	Name	Dimensions	Flow rate & injection vol.	Mobile phase & Elution
Size-exclusion (SEC)	2 GE Healthcare SEC columns in series, i.e., Superdex 75 Increase and Superdex Peptide 10/300 GL	10 × 300 mm, 13 μm & 10 × 300 mm, 5 μm (used with in-line filter)	1 mL min^− 1^ 100 µL	5 mM Ammonium nitrate
Mixed-mode	Thermo Fisher Scientific: Hypercarb	100 × 4.6 mm, 5 μm (with guard column)	1 mL min^− 1^ 100 µL	80% 240 mM formic acid, 1%MeOH, pH 2.4 (isocratic)
Cation-ex.	Agilent: IonospherC	100 × 3 mm, 5 μm (with guard column)	1 mL min^− 1^ 100 µL	0.75–8 mM pyridine formate, 3% MeOH, pH 3–3.2 (gradient)
Anion-ex.	Hamilton: PRP-X-100	100 mm x 4.1, 5 μm (used with in-line filter)	1.25 mL min^− 1^ 100 µL	1.6–10mM ammonium citrate, pH 5.2, 2% MeOH (gradient)

For the mixed-mode, cation-exchange and anion-exchange analysis, peaks (^78^Se) were integrated using the software MassHunter and Se speciation in samples was calibrated using compound independent calibration. Compound independent calibration was performed using Se(IV), except in the case of the IonospherC column where Se(IV) elutes close to the column void, hence in this case the average of the slopes of the two commercially available organic Se species SeMet and MeSeCys (Sigma-Aldrich) was used. In case important drifts in instrument sensitivity were noted in between samples (changes in average Y counts per second (cps) > 10%), the Se signal was corrected by average Y counts throughout the chromatogram for every sample. Since in the case of the SEC analysis a more diverse set of Se-containing compounds was expected in the samples (SEC analysis included SDS extracts likely containing selenoproteins, while SDS extracts were not injected into the mixed-mode, cation-exchange and anion-exchange column), we opted for Se quantification via on-line post-column isotopic dilution (infusing ^78^Se(IV) post-column and monitoring the 78/80 Se ratio as detailed in Tolu et al., 2022), instead of compound independent calibration.

### Data Analysis and Statistics

Peak deconvolution of the calculated Se mass flow in all samples was performed with the software Origin (OriginPro) while all other data analysis was made with RStudio (R version 4.4.2). Data are presented as mean ± SD where SD refers to the standard deviation between three extraction replicates in parts 3.1–3.3 of the manuscript while in part 3.4 (Se exposure experiments) it refers to biological replicates. Observed differences between extraction techniques were tested for statistical significance using one-sided Welch’s t-test (α = 0.05) when testing whether successively added treatment steps resulted in increases in extraction relative to the control; differences in photosynthetic yields of Se exposure experiments were assessed using two-sided student’s t-test (α = 0.05).

## Results and Discussion

### How to Harvest, Wash and Extract?

#### Cell Harvesting and Washing

We first investigated the effects of cell harvesting and washing, prior to extraction, on the quantification of intracellular Se. The goal was to minimize the presence of Se(IV) from the growth medium which may be extracellularly bound or carried-over from residual cell medium contained in the cell pellet - whilst minimizing losses of intracellular Se species. Therefore, we quantified putative Se(IV) (through RT matching with standard addition), as well as additional unidentified organic Se species, in *G. huxleyi* and *E. voratum* after different harvesting and washing protocols (Fig. S2). In both algae, Se(IV) concentrations in the final cell extracts were strongly reduced by increasing the number of cell wash cycles, and further by including PO_4_^3−^ in the washing solution. The concentrations of organic species on the other hand remained largely stable, indicating that the washing procedure successfully separates intracellular from extracellular Se. Harvesting cells by centrifugation was superior to filtration, as filtered cells consistently showed higher Se(IV) concentrations that could not be reduced by further washing (e.g. Se(IV) concentrations in *G. huxleyi* after 3 washes with PO_4_^3−^ containing washing solution = 1.8 ± 0.2 µg L^− 1^ (with filtration) or 0.22 ± 0.02 µg L^− 1^ (with centrifugation)). In light of this finding, data presented thereafter are from cells harvested by centrifugation and washed 3 times with an isotonic NaCl buffer containing 25 mM PO_4_^3−^.

#### Total Se Extraction Efficiencies

**Fig. 1:**
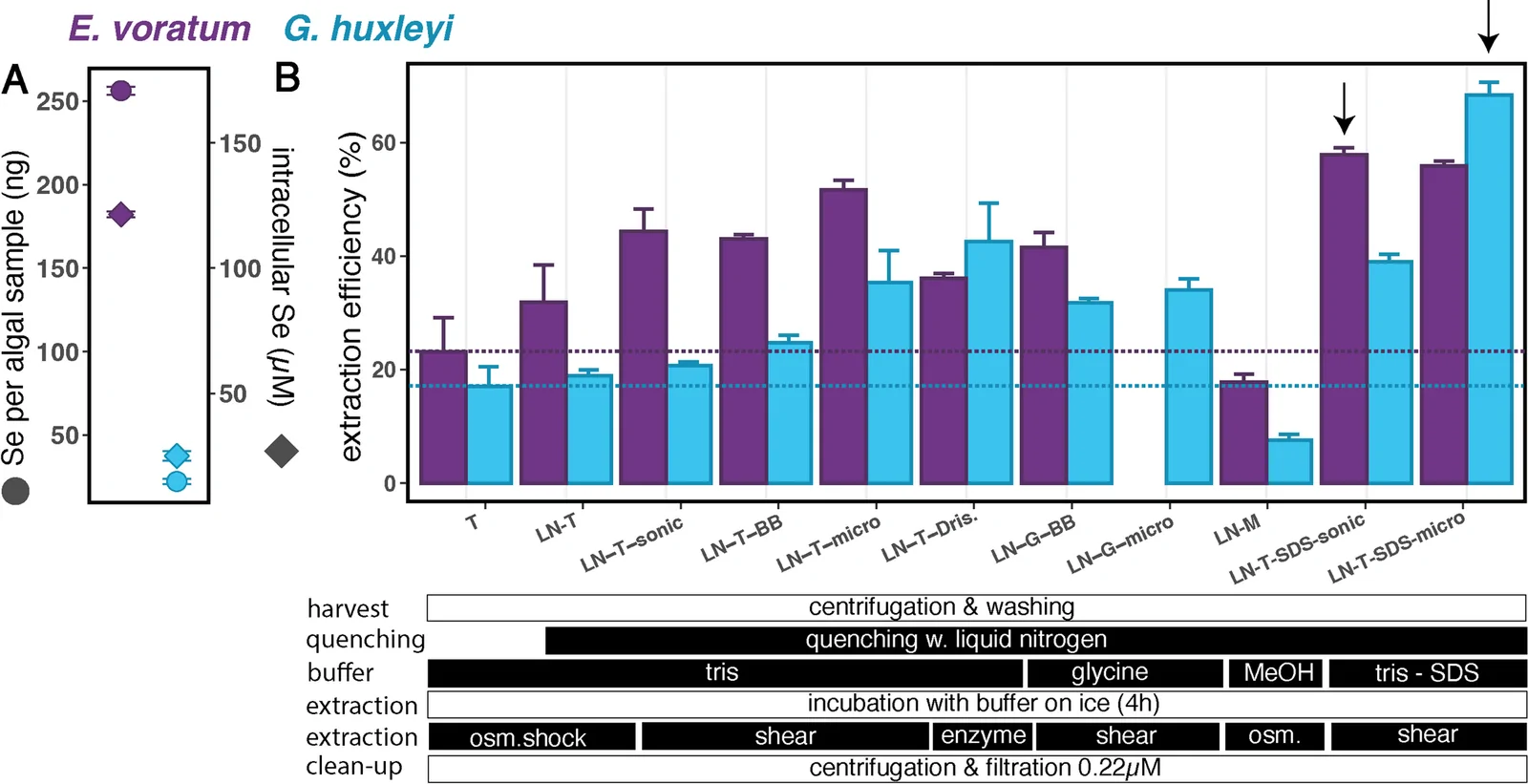
**A** Circles: Total intracellular Se loads in an algal sample of *E. voratum* (purple) and *G. huxleyi* (blue) when harvesting 100 mL of a stationary phase culture grown at 100 µg L^-1^. Diamonds: intracellular Se concentrations accounting for cell counts and size. **B** Corresponding Se extraction efficiencies for the various protocols tested (LN: liquid nitrogen, M: MeOH, T: Tris-HCl buffer, G: Glycine buffer, BB: cryogenic bead beating, Micro: microwaving, sonic: sonication, Dris: enzymatic driselase extraction). Extraction efficiencies were determined by dividing the total Se measured in digested cell extracts by the total Se measured in digested, unextracted cell pellets. Dotted lines mark reference extraction efficiencies of the control treatment (incubation of cells in Tris without further treatments), stars show treatments with the highest extraction efficiencies achieved. Note that no sample was available for *E. voratum* "LN-G-micro"

We tested 11 different protocols for extracting Se from the two microalgae species *G. huxleyi* and *E. voratum*, consisting of varying quenching and cell lysis techniques using various extraction media. The performance of these protocols was first assessed based on total Se extraction efficiencies (presented here) and then based on relative differences in Se speciation in resulting extracts (presented in following sections). *E. voratum* whole cell pellets were higher in total Se than those of *G. huxleyi*, containing 256 ± 2 ng and 22 ± 2 ng Se, respectively (circles in Fig. 1A), translating to intracellular Se concentrations of 121 ± 1 µM and 27 ± 2 µM in the two algae when taking into account cell counts and biovolume estimates (diamonds in Fig. 1A).

We hypothesized that incubation in Tris buffer alone (“T”), without shear stress, may create hypo-osmotic conditions relative to seawater media, potentially leading to partial release of intracellular compounds via osmolyte efflux or osmotic lysis [70, 71]. However, susceptibility to osmotic lysis is likely taxon-dependent, as cell coverings vary widely in structure and composition between phytoplankton species [72] and may partially protect against rupture. Consistent with limited lysis under these conditions, this extraction (T) released the lowest amount of Se of any aqueous extraction for both algae (*E. voratum*: 23 ± 6%, *G. huxleyi*: 17 ± 3%) (Fig. 1B). Quenching cells with liquid nitrogen prior to extraction in Tris buffer (“LN-T”) generally increased extraction efficiencies for *E. voratum* and was therefore included in all other treatments, even though the increase was not statistically significant (*p* = 0.08) and absent for *G. huxleyi*. Inducing additional cell disruption through shear stress via either sonication or bead beating (“LN-T-sonic” and “LN-T-BB”, respectively) significantly increased extraction efficiencies in both algae (*p* < 0.05 in all cases). Microwaving (“LN-T-micro”) even further increased extraction efficiencies, reaching a maximum of 52 ± 2% for *E. voratum* and 35 ± 6% for *G. huxleyi*. Degrading the algal cell wall biologically instead of mechanically using driselase, an enzymatic cocktail containing a mixture of cell-wall degrading enzymes like cellulase and hemicellulose (“LN-T-driselase”), proved to be superior than microwaving for *G. huxleyi* but not *E. voratum*. Despite the high extraction efficiency reached, we caution against the use of driselase unless strictly required since the material itself contains a high Se load (148 µg Se kg^− 1^ driselase), making it not suitable for low concentration samples and requiring rigorous blank corrections.

We further investigated the effect of changing the extraction solution from neutral pH Tris (pH 7.5) to an acidic glycine (“G”) buffer (pH 2.5). This had no significant effect for *E. voratum* and only showed minor improvements for *G. huxleyi* when used in combination with bead beating but not with microwaving. We also tested the organic solvent methanol (MeOH, “M”) which is traditionally employed in metabolomic workflows, after extraction MeOH extracts were evaporated and the residue was resuspended in Tris buffer. This protocol yielded the lowest extraction efficiencies recorded in this study, i.e. 18 ± 2% and 9 ± 1% in *E. voratum* and *G. huxleyi*, respectively. We found no major differences in MeOH extracts dried using a vacuum centrifuge vs. using nitrogen (dried either fully or to incipient dryness) when characterized using mixed-mode chromatography (data not shown).

The addition of SDS, expected to solubilize Se-proteins, to the extraction buffer (Tris) significantly improved extraction efficiencies for both algae (*p* < 0.05 for all pairings, i.e. micro & sonic with and without SDS for both algae) and achieved the highest extraction efficiencies recorded in this study (*E. voratum* 58 ± 1% “LN-T-SDS-sonic”; *G. huxleyi* 68 ± 2% “LN-T-SDS-micro”). In the case of *G. huxleyi*, microwaving together with SDS led to significantly (*p* < 0.05) better results than sonication with SDS, however this difference was not found for *E. voratum*. These extraction efficiencies agree well with what has been previously described for natural microphyte samples from agricultural wetlands (43–89%, extracted using an SDS-based protocol as well) [9].

Overall, we conclude that while common in metabolomics and useful for its enzyme quenching and protein precipitating function, cold MeOH extractions were not suitable for our purpose. Instead, aqueous extractions with Tris buffer aided by mechanical shear stress and the addition of SDS resulted in the highest extraction efficiencies for our two model marine phytoplankton species, even though about a third of algal intracellular Se still remains inaccessible for (liquid-based) analysis.

### Which Chromatographic Techniques for Which Research Questions?

#### Size-Exclusion Chromatography for LMW vs. HMW Se Species

##### Full Recovery of Extracted Se with SEC-UV-ICP-MS/MS

**Fig. 2:**
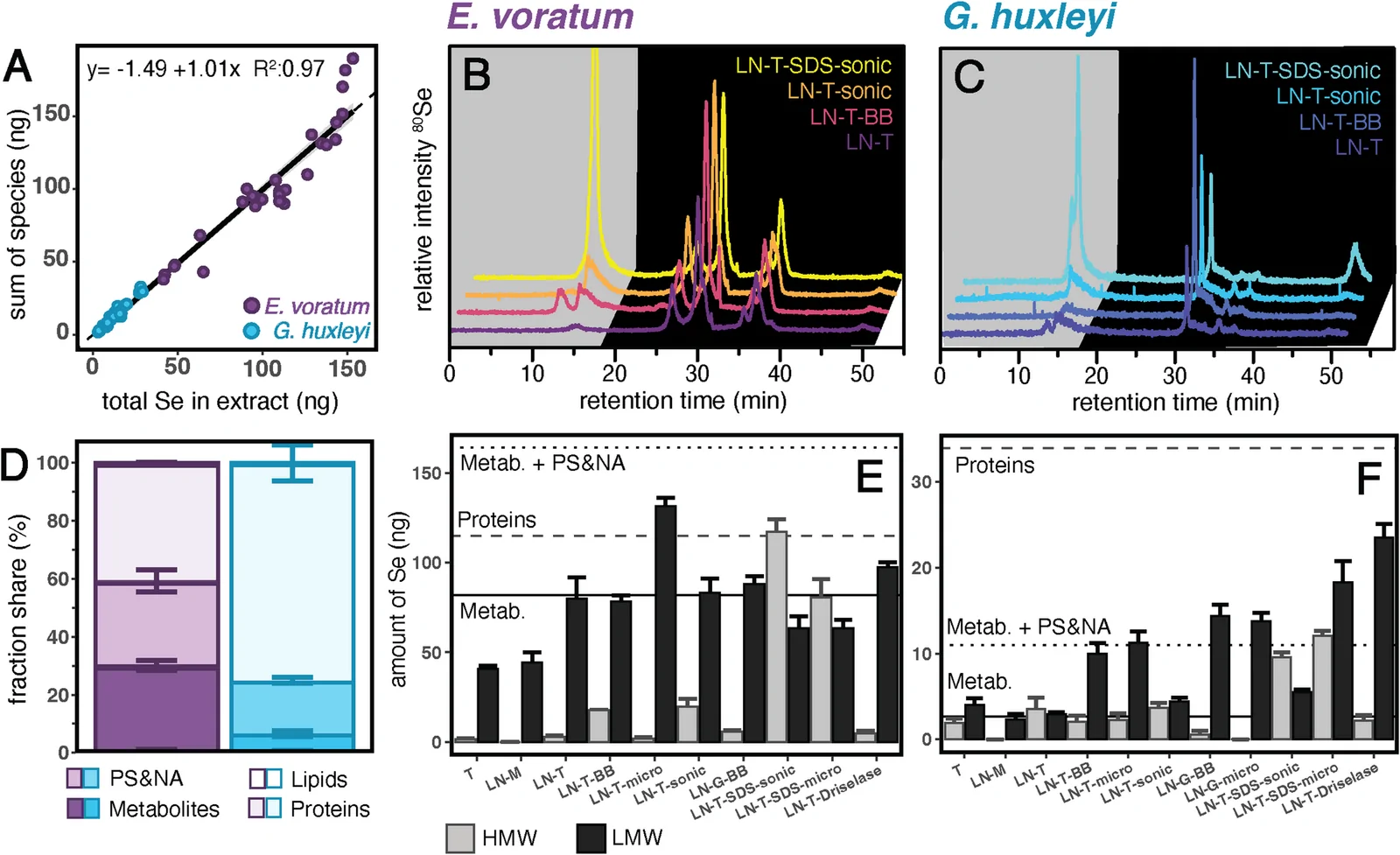
**A**: Column recovery of the SEC column, showing the total amount of Se (x axis) versus the sum of SEC species quantified by post-column isotopic dilution (y axis). **B** & **C**: Representative SEC-ICP-MS/MS chromatograms for selected extraction conditions **D**: Relative distribution of Se across cellular components, operationally defined according to Obata et al., 2004. Note that Se in the lipid fraction was negligible (< 2%). **E** & **F**: Amounts of Se in high molecular weight (HMW, eluting < 18 min) and low molecular weight (LMW, eluting > 18 min) Se species. In all cases error bars represent standard deviations of three extraction replicates. Lines show the total amount of Se (ng per sample) present as metabolites (solid), or as the sum of metabolites and polysaccharides & nucleic acids (PS&NA) (dotted). Dashed lines show the amount of Se present as proteins (all determined by sequential extractions, relative distribution shown in **D**)

To examine the size distribution of Se compounds in the cell extracts we employed size-exclusion chromatography (SEC) using 2 columns in series, obtaining a separation of compounds up to ∼70 kDa. The combination of these SEC columns with on-line isotopic dilution (post-column addition of ^78^Se(IV)) allowed for precise Se quantification in SEC peaks, resulting in complete column recoveries across all extraction protocols for both algae species used in this study (*E. voratum* 97 ± 12% (*n* = 30), *G. huxleyi*: 95 ± 18% (*n* = 31), Fig. 2A).

Typical SEC chromatograms for selected cell extracts of both *G. huxleyi* and *E. voratum* are displayed in Fig. 2B and C. These samples generally show two distinct groups of Se-containing compounds that are present in both algae species, albeit in different proportions, with the first group eluting before 18 min (grey underlay in Fig. 2B & C) and the second after 25 min (black underlay). Based on our size calibration (see supplementary note S4) and the observation that the addition of SDS, an anionic detergent traditionally employed to solubilize proteins, selectively increased only the first group of compounds (RT < 18 min), we here defined these compounds as high molecular weight (HMW) (MW > 44 kDa according to the protein standards tested) and compounds eluting after 18 min as low molecular weight (LMW) compounds. This classification is further supported by the observation that the compounds with retention times (RT) < 18 min - which increased upon SDS addition - contained Fe, Co, Cu, and Zn (see Fig. S5 and S6). We hypothesize that these metal-containing compounds correspond to metalloproteins. We however note that the small protein size standards aprotinin, myoglobin and cytochrome for example elute with RT between 25 and 27 min and would hence erroneously be classified as “LMW” by our selected cutoff for these SEC analyses. Further discussion on the capacity of this SEC column combination to separate Se containing compounds according to size can be found in supplementary note S4.

##### Effect of Extraction Protocols on HMW vs. LMW Se Distribution

The various extraction protocols tested in this study produced extracts with a wide range of relative distributions between LMW and HMW Se-containing fractions (Fig. 2E & F). Extracting both HMW and LMW species at once could either be valuable for maximizing the information gained with a single extraction but also deleterious for workflows targeting LMW Se-metabolites using small-bore HPLC columns that may be damaged by injecting HMW species.

Expectedly, adding SDS to the extraction buffer greatly increased the extraction of HMW-Se (likely Se-protein), while still extracting comparable amounts of LMW-Se as Tris-only (i.e. no shear stress) extractions. On the other hand, shear stress (BB or micro) or the addition of driselase selectively increased the extraction of LMW Se-compounds compared to buffer-only extracts, especially in *G. huxleyi*; however, relatively high amounts of HMW-Se were still detectable in these extracts. Effective prevention of HMW-Se extraction could only be achieved by extracting with MeOH, which also significantly lowered extraction of LMW-Se for *E. voratum* (*p* < 0.05) (decrease not significant for *G. huxleyi* (*p* = 0.11)), or glycine buffer, which reduced HMW-Se extraction while maintaining or improving LMW-Se extraction compared to Tris buffer (e.g. *E. voratum* LN-G-BB vs. LN-T-BB LMW: 88 ± 4 ng vs. 78 ± 3 ng, HMW: 5.8 ± 0.6 ng vs. 17.6 ± 0.3 ng; *G. huxleyi*: LN-G-BB vs. LN-T-BB LMW: 14 ± 1 ng vs. 10 ± 1 ng, HMW: 0.6 ± 0.4 ng vs. 21 ± 0.7 ng). Therefore, the LN-G-BB protocol provided the best combination of high extraction of LMW and low extraction of HMW Se in preparation for analyses of LMW Se metabolites using other, small-bore analytical columns.

##### Sequential Extractions in Comparison to Sec Results

The distribution of Se in the different biochemical fractions (i.e., HMW proteins, polysaccharides & nucleic acids, lipids, and LMW metabolites) of the two algae strains is presented in Fig. 2D. Closed mass balances between the sum of all chemical fractions and total digestions of unfractionated algal cell pellets demonstrate that no Se was lost during the fractionation protocol (recoveries: 109 ± 5% for *E. voratum*, 110 ± 6% for *G. huxleyi*).

We attempted to compare information gained about intracellular Se distribution in these different biochemical fractions between sequential extractions and SEC-ICP-MS/MS analyses. Sequential extractions showed higher relative proportions of Se in proteins in *G. huxleyi* (75 ± 6%) compared to *E. voratum* (40.8 ± 0.1%) under these growth conditions, this result for *G. huxleyi* agrees well with a previous quantification of Se in proteins for a different strain (NIES 873) [11]. Higher relative proportion of Se in proteins in *G. huxleyi* are in line with the generally higher abundance of HMW-Se in non-SDS extracts of *G. huxleyi* (20 ± 19%) compared to *E. voratum* (7 ± 7%), as determined by SEC-ICP-MS/MS. However, upon SDS addition and sonication, a lower share of HMW-Se was additionally solubilized from *G. huxleyi* in comparison to *E. voratum*, yielding relative HMW-Se shares of 51 ± 13% and 60 ± 6%, respectively. Figure 2E and F show how the Se-protein quantification via sequential extractions (indicated by dashed lines) compares to that of the SEC analysis (indicated by grey bars), assuming that the whole HMW-Se fraction represents Se-proteins. Under this assumption, Se-protein recovery in SDS extracts measured by SEC-ICP-MS/MS was fair for *E. voratum* (80 ± 10 or 117 ± 7 ng HMW Se quantified by SEC in SDS-sonic and SDS-micro extracts vs. 114.9 ± 0.7 ng Se in proteins quantified by sequential extraction) but poorer for *G. huxleyi* (9.6 ± 0.6 and 12.1 ± 0.6 ng HMW Se quantified by SEC SDS-sonic and SDS-micro extracts vs. 34 ± 3 ng Se in proteins quantified by chemical fractionation).

Sequential extractions also elucidated the potential nature of LMW-Se extracted in SEC-ICP-MS/MS analyses (black bars in Fig. 2E & F). Cells quenched with LN and extracted with only Tris buffer (“LN-T”) showed LMW-Se amounts comparable to amounts of Se-metabolites from sequential extraction (sequential extraction LMW Se: 30 ± 2% of total intracellular Se in *E. voratum* and 9 ± 2% in *G. huxleyi*), indicated by dotted lines in Fig. 2E & F. Adding shear stress increased the extracted LMW-Se amounts to be on par with combined amounts of Se-metabolites, -polysaccharides, and -nucleic acids (sequential extraction PS&NA, 29 ± 4% of total intracellular Se in *E. voratum* and 18 ± 1% in *G. huxleyi*; indicated by dotted lines in Fig. 2E & F). This increase in LMW-compound extraction could be due to breakdown of such PS&NA macromolecules into soluble, more easily extractable, fragments upon shear stress. Overall, these results demonstrate that SEC analyses can be a useful tool to deduce information about the intracellular Se distribution between HMW and LMW metabolite fractions in microalgae without performing sequential extractions.

#### Other Chromatographic Techniques to Differentiate LMW Se Species

**Fig. 3:**
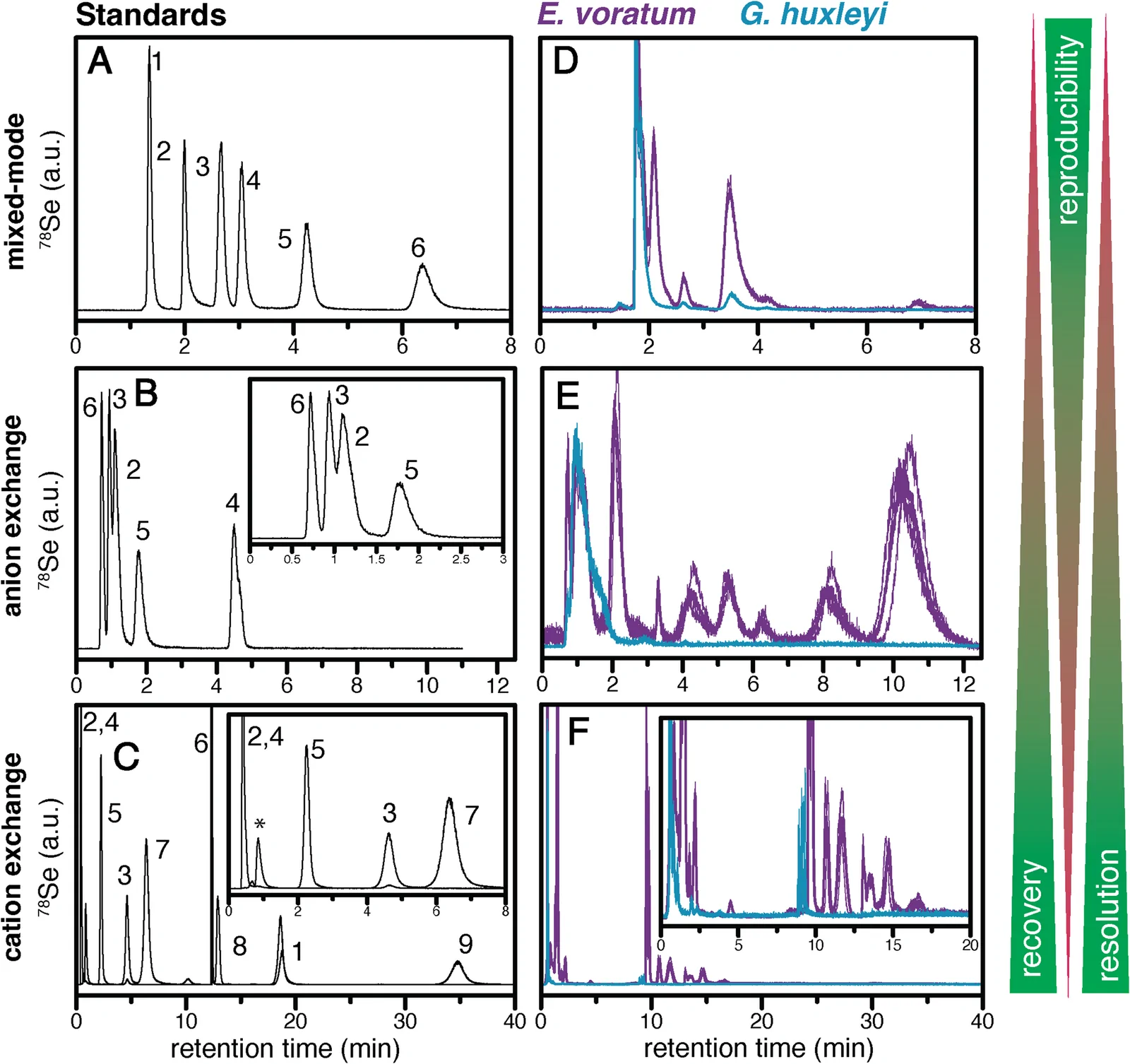
Chromatograms of standards (left panels) and algal extracts (right panels) for the three analytical columns tested in this study. Thin lines in** D**, **E** and **F** show analytical extraction replicates, thick lines represent the average of the three chromatograms. Algal extracts were obtained by centrifugation and subsequently washed with a PO_4_^3−^ containing isotonic NaCl buffer, quenched in liquid nitrogen, resuspended in Tris-HCl, and cryo-milled (LN-T-BB). Standards: 1 – TMSe, _2_ – SeIV, 3 – MeSeCys, 4 – SeVI, 5 – SeMet, 6 – SeCys2, 7 – SeEt, 8 – DMSeP, 9 – MeSeMet. Note that even though the separation between methylselenocysteine (MeSeCys) and Se(IV) on the anion-exchange column is poor, we included both standards in the calibration curves since Se(IV) is the form of Se we provide to algae during cultivation and MeSeCys is one of the key suspected metabolites in their extracts. SeEt (7) was later found to also elute on anion exchange and mixed-mode shortly after Se(VI) (4) and after SeCys_2_ (6), respectively

We further investigated what mixed-mode, anion-exchange and cation-exchange chromatography can reveal about LMW Se speciation in our two model phytoplankton species. Figure 3 (A**-**C) shows the elution of LMW Se standards with the selected methods, as well as typical chromatograms obtained from *E. voratum* and *G. huxleyi* Tris-based extracts (D**-**F). An additional discussion about LMW Se standard retention by the selected methods can be found in SI Note 3.

##### Column Resolving Power and Reproducibility

**Fig. 4:**
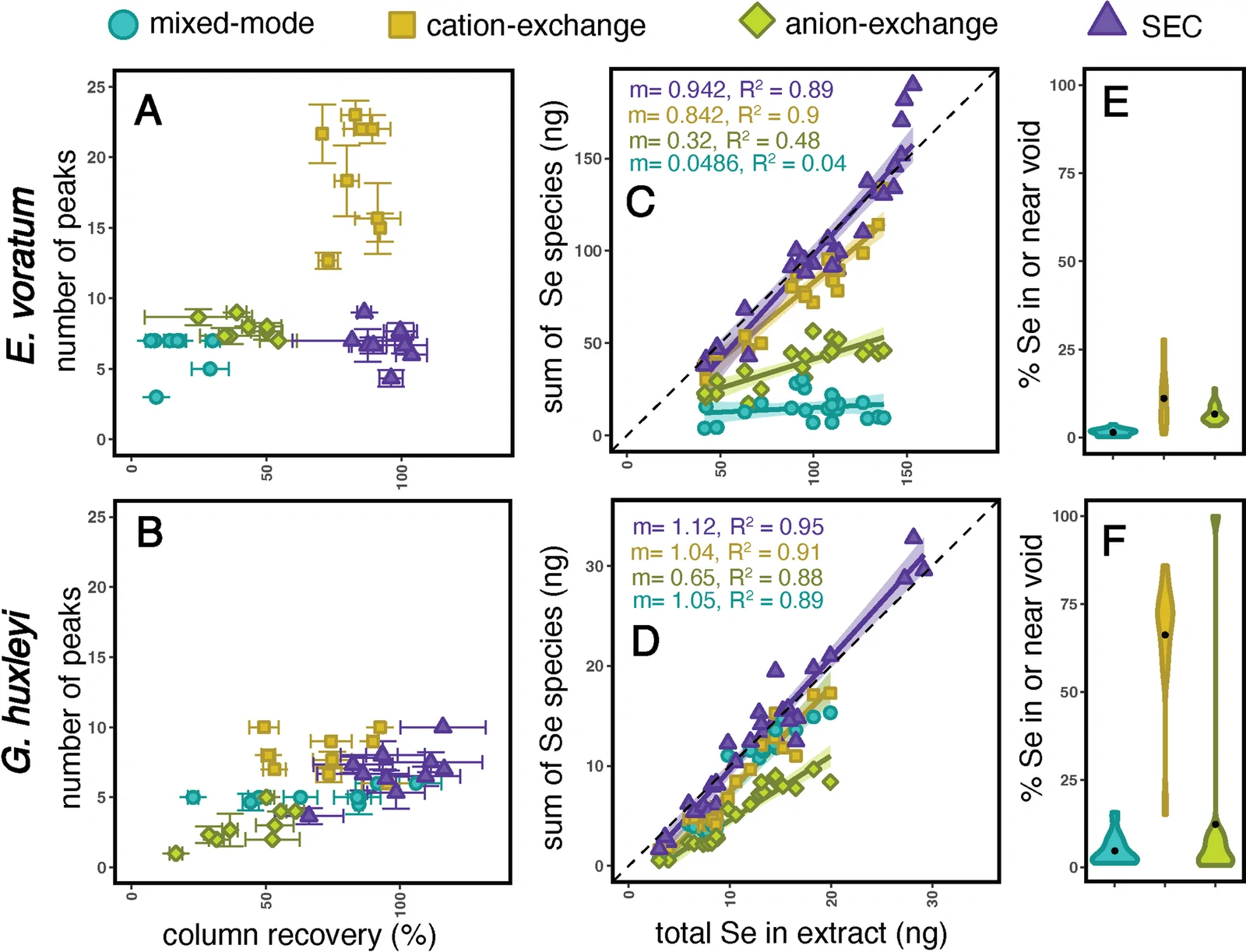
**A** & **B**: Number of distinct peaks vs. recoveries of the various columns (mixed-mode Hypercarb column, the cation-exchange IonosphereC column, the anion-exchange PRP-X-100 column or the size-exclusion Superdex peptide columns (SEC) for extracts of *G. huxleyi* and *E. voratum* prepared using all extraction protocols. Error bars show standard deviations of extraction replicates. **C** & **D**: Column recovery analysis, showing total Se measured in extracts (x axis) vs. the sum of Se species eluting from the respective column, quantified by isotope dilution for the SEC and by compound independent calibration for the remaining three columns. **E** & **F**: Share of Se eluting in or near the void for each column

Extracts from both algae showed 4–5 main Se peaks on the mixed-mode column (Fig. 3D), the majority of which eluted between 2 and 4 min and are similar in RT between extracts from *G. huxleyi* and *E. voratum*. The mixed-mode column showed the highest chromatographic reproducibility under our testing conditions, i.e., deviations in retention time between injected analytical extraction triplicates were minimal, as was also the case for the SEC column (see extraction replicates in Fig. 3). The anion-exchange column on the other hand excelled at resolving differences between extracts of the two algal species: while *G. huxleyi* and *E. voratum* extracts looked very similar in terms of chromatogram profiles on the mixed-mode column, these samples look extremely different after separation by anion-exchange chromatography (Fig. 3E). *G. huxleyi* extracts (all replicates) exhibited two main poorly separated peaks with RT < 2 min, contrasting *E. voratum* extracts which eluted as well separated, large Se peaks within 8–9 min (Fig. 3E). We assume these species to be too hydrophobic to elute from the mixed-mode column under our chromatographic conditions, rationalizing why particularly low column recoveries were observed (see below, Sect. 3.2.2). Comparing Se speciation between *E. voratum* and *G. huxleyi* using the mixed-mode column only would therefore have erroneously suggested that the Se-metabolomes of these two phytoplankton species are similar. The anion-exchange column also showed relatively high chromatographic reproducibility, albeit lower than the mixed-mode one. While the anion-exchange column showed to be well suited for separating Se compounds contained in *E. voratum*, more information could be gained about *G. huxleyi* extracts when analysed with the cation-exchange column (Fig. 3F). In this case, 6 to 10 peaks were detected for the *G. huxleyi* extracts, though nonetheless the majority of Se eluted within a single peak in or near the column void (Fig. 3F). For *E. voratum* extracts, the cation-exchange column showed outstanding chromatographic resolving power with up to 24 Se peaks detected in extracts and only a minor fraction eluting in or near the column void (15 ± 9% across all treatments, Fig. 4E). These peaks were found in two main regions of the chromatogram: (1) between the column void and 5 min and (2) between 10 and 18 min (see also Fig. S8 for a zoom on the first phase). On the downside, chromatographic reproducibility of this column tended to be low and substantial shifts in RT occurred for *E. voratum* (but not *G. huxleyi*) extracts. Even though the reproducibility of replicate injections generally improved over the course of a run, RT shifts remained especially between 10 and 20 min (Fig. 3F). Therefore, despite very long column acclimatization times with the mobile phase prior to analysis, reproducibility always remained lower than the mixed-mode or anion-exchange columns. While all columns showed matrix effects for extracts containing buffers (Fig. S9), i.e., resulting in changes in RT and peak shape, these effects were strongest on the cation-exchange column where especially the presence of glycine buffer in the sample strongly affects elution of standards.

##### Column Recoveries

On top of chromatographic reproducibility and resolving power, column recovery is a key parameter when working with natural samples containing unknown chemical species. In addition to the low resolving power described above, the mixed-mode column generally had very low column recoveries (Fig. 4C & D). While *E. voratum* extracts contained total amounts of Se between 42 and 153 ng per cell pellet, only 0.7 to 15 ng eluted out of this column, translating into recoveries between 7.2 ± 0.2% and 30 ± 3% (average of 17 ± 9%). There was no significant relationship between the total Se content in extracts and the amount of Se eluting from this column (R^2^ = 0.04). While column recoveries overall were much higher for *G. huxleyi* extracts (average of 64 ± 20%), it was also extremely variable based on extraction technique and not representative of the total amount of Se present in the sample (min. 27 ± 4% for LN-M and max. 85 ± 4% for LN-G-micro). The anion-exchange column despite revealing striking differences between *G. huxleyi* and *E. voratum* extracts with up to 10 well separated chromatographic peaks in the latter (Fig. 4B), also showed relatively low column recoveries for both species (average of 43 ± 9% and 43 ± 14% for *E. voratum* and *G. huxleyi*, respectively) but the amount of Se eluting from the column was in both cases positively (R^2^ of 0.67 and 0.88) correlated with the total amount of Se in extracts. The cation-exchange column showed the highest column recoveries out of these three columns for both algae species with averages of 73 ± 17% and 83 ± 8% for *G. huxleyi* and *E. voratum* across all treatments tested. We found that in aqueous *G. huxleyi* extracts, low column recoveries are strongly related to the presence of HMW Se species for all three columns (R^2^ of 0.92, 0.9 and 0.96 on mixed-mode, cation-exchange and anion-exchange, respectively) (Fig. S10). However, this correlation did not materialize in aqueous *E. voratum* extracts, or in the methanol-based extracts of either algae – these extracts showed particularly low column recoveries despite the complete absence of HMW Se species. This illustrates that the molecular weight of organic Se compounds is an important, but not the only, factor affecting column recovery and that other parameters such as charge and hydrophobicity must play a role.

In conclusion, all columns tested in this study offered unique benefits but also downsides for the analysis of Se in marine phytoplankton extracts. Most importantly, we found that the characterization of an algal selenometabolome can change substantially depending on which analytical column was used. Therefore, rigorous verification of column recoveries and the use of multiple analytical columns is key for these complex samples. If the primary goal of a study lies in qualitatively comparing and uncovering differences in LMW Se compounds between different algae species, anion-exchange chromatography is a promising tool. If targeted, quantitative comparisons of specific Se-species are desired, mixed-mode chromatography will yield most robust results. If quantitative comparisons including HMW species are required, the SEC column would be appropriate. Finally, cation-exchange chromatography appeared too unreliable in terms of RT to compare Se species between different matrices but nevertheless provides excellent separation efficiency and column recoveries. These latter features make cation-exchange chromatography a promising tool for projects aiming at untargeted, novel identification of Se compounds using HPLC coupled to molecular high resolution tandem mass spectrometry (HR-MS/MS) in which case stable RT are less critical than for analysis by HPLC-ICP-MS/MS.

### Does the Choice of Extraction Technique Affect LMW Se Speciation?

**Fig. 5:**
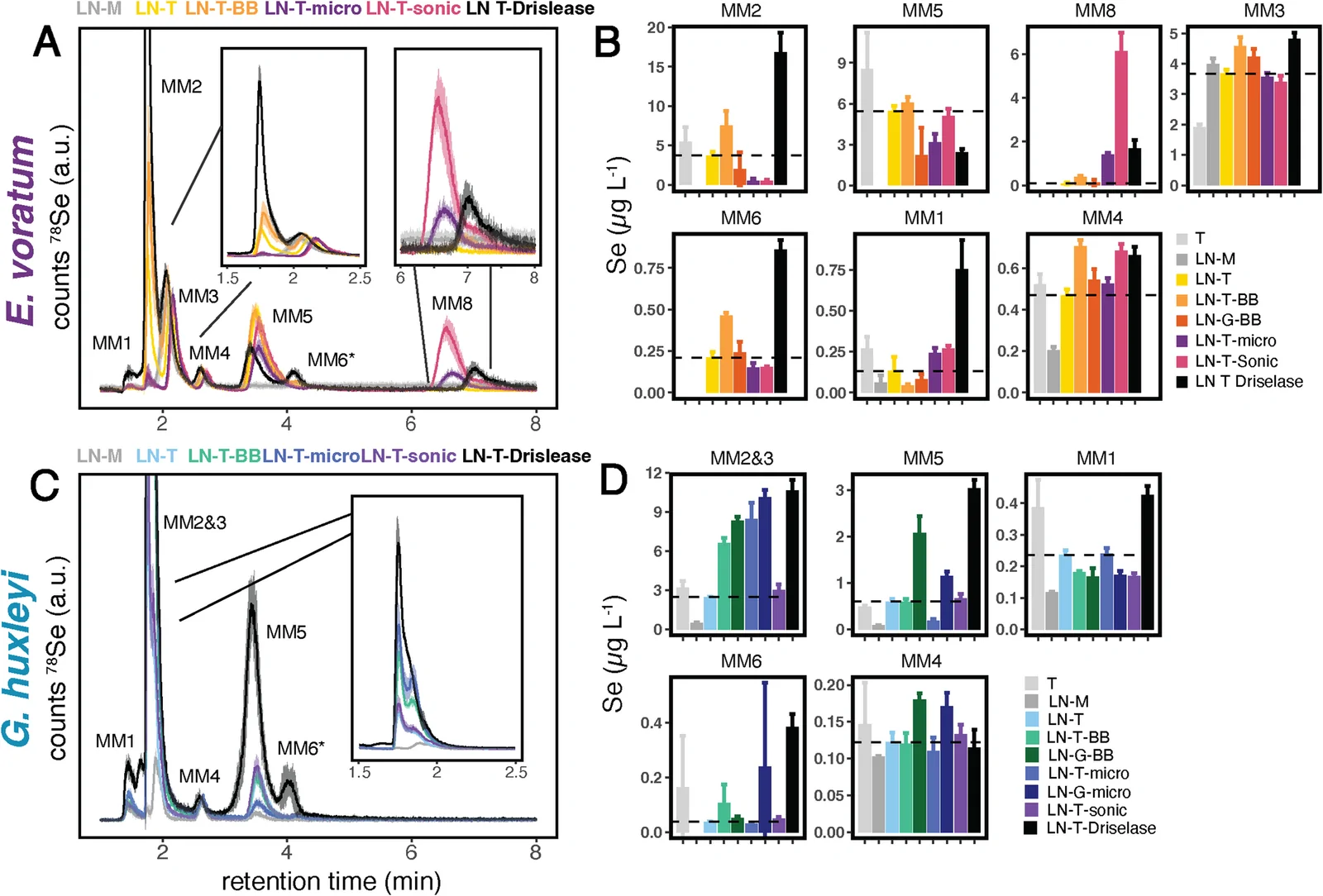
Effect of extraction conditions on Se speciation in *E. voratum* (**A** & **B**) and G. huxleyi (**C **& **D**) (grown at 100 µg L^-1^ Se) cell extracts as measured by mixed-mode chromatography. **A** & **C**: Example chromatograms illustrating specific changes in chromatogram shape with different treatments. Solid lines show the average of three chromatograms with the shaded areas showing chromatogram standard deviations (extraction replicates). MM6* is also contained in the Driselase blank and hence needs to be subtracted. **B** & **D**: Comparison of integrated peaks, black dotted lines show the average concentration of the respective peak in the treatment "LN-T". "MM2-8" refer to the respective peaks visible on the mixed-mode column

Beyond affecting total yields and relative distributions of LMW vs. HMW species, extraction conditions might impact the speciation of LMW Se in phytoplankton extracts. We investigated such extraction-induced changes in Se speciation using the mixed-mode column (Fig. 5), which was the most robust for such comparisons due to its high reproducibility, as well as the anion-exchange column (see Fig. S11). Using the mixed-mode column, the Se peaks observed (which we note could represent mixtures of different, co-eluting Se species) were largely the same in RT but their intensities varied based on the extraction technique; this was the case for both tested algae. Certain peaks showed a clear response to the type and magnitude of shear stress applied to the sample (e.g. in *E. voratum*, peaks at RT 1.8 (MM-2) and 7–7.5 min (MM-8), see also Fig. S12). In contrast, other peaks (RT 3.8 (MM-5), also MM-2, MM-8) were no longer detectable when samples were extracted with MeOH. We cannot determine whether this reflects poor solubility of compounds in MeOH, degradation during extraction or evaporation (samples were reconstituted in aqueous phase prior to injection, see M&M) or suppression of enzymatic processes that may generate these LMW Se compounds. Finally, we investigated for *E. voratum* to what extent the first extraction recovers all, or only a fraction of the extractable organic Se species by repeating the complete extraction protocol a second time on the cell pellets remaining from the first extraction. No matter the extraction technique used, the second extraction in all cases produced samples with only a single Se peak, MM-2 (RT match with Se(IV)), and none of the (other) organic Se species (Fig. S13), hence a single extraction appears to be sufficient to extract the majority of LMW organic Se species.

In conclusion, we can demonstrate that the choice of extraction technique not only affects total Se yields, but also the molecular weight distribution of Se species (observed using SEC), and the recovery or relative extracted amounts of specific LMW Se species (observed using MM chromatography). Nevertheless, the observed changes in chromatograms between treatments are minor compared to differences observed between the two algae species selected for this study.

### What is the Effect of Increasing Medium Se Concentrations on Se Speciation Studies in Marine Algae?

Lastly, we tested to what extent increasing the concentration of Se, supplied as Se(IV), in the growth medium affects experimental studies of Se speciation in marine algae, including effects on growth and fitness of cells, as well as their selenometabolism.

#### Impact of High Se on Algal Growth and Fitness

**Fig. 6:**
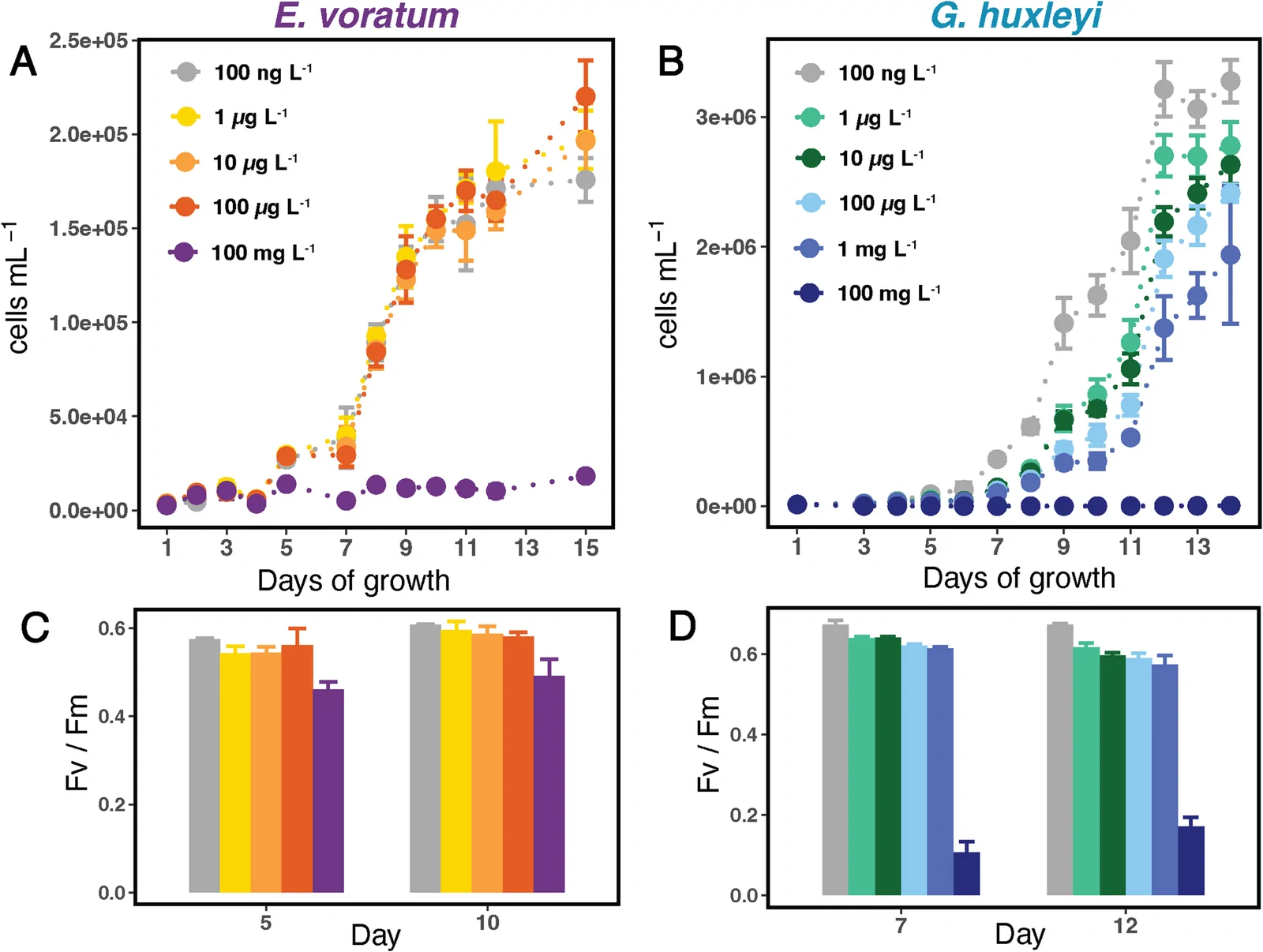
Growth curves and photochemical efficiency measurements of *E. voratum* (**A** & **B**) and *G. huxleyi *(**C** & **D**) grown at different Se(IV) medium concentrations (represented as different colors). Cell counts in cells mL^− 1^ were determined by flow cytometry, photochemical efficiency values (ratio of variable fluorescence (Fv) to maximum fluorescence (Fm)) were determined by PAM-fluorometry

Exposing *E. voratum* and *G. huxleyi* to increasing levels of medium Se(IV) resulted in substantial effects on algal growth and fitness in both species, with *E. voratum* being relatively more tolerant to high Se levels than *G. huxleyi* (Fig. 6). For *E. voratum*, cell growth remained relatively stable between 0.1 and 100 µg Se L^− 1^ but dropped dramatically when further increased to 100 mg Se L^− 1^, concomitant with a small but significant (*p* < 0.05) decrease in photochemical efficiency (Fig. 6B). Because of this strong response, we introduced an additional concentration level of 1 mg Se L^− 1^ for *G. huxleyi*. Here, we observed a more pronounced and gradual response to increasing medium [Se(IV)]. Both biomass yield as well as photosynthetic efficiency decreased significantly (*p* < 0.05) between 0.1 and 1 µg Se L^− 1^. These effects intensified with further increases in medium [Se(IV)] (Fig. 6C & D). At 100 mg Se L^-1^, *G. huxleyi* cell counts were reduced by 99.9% relative to 0.1 µg Se L^-1^, concomitant with a strong decrease in Fv/Fm. These cells showed strong orange discoloration which we speculated to be caused by precipitation of intracellular elemental Se(0), as previously described for other algae, e.g. *C. rheinhardtii*, under excessively high medium Se(IV) concentrations [19]. These *G. huxleyi* pellets grown at 100 mg Se L^− 1^ were therefore not used for any further Se analysis as the detection of Se(0) – or other, artificial, high medium Se induced compounds - was not the objective of this work.

#### Changes in Se Bioconcentration Factors and Extraction Efficiency

**Fig. 7:**
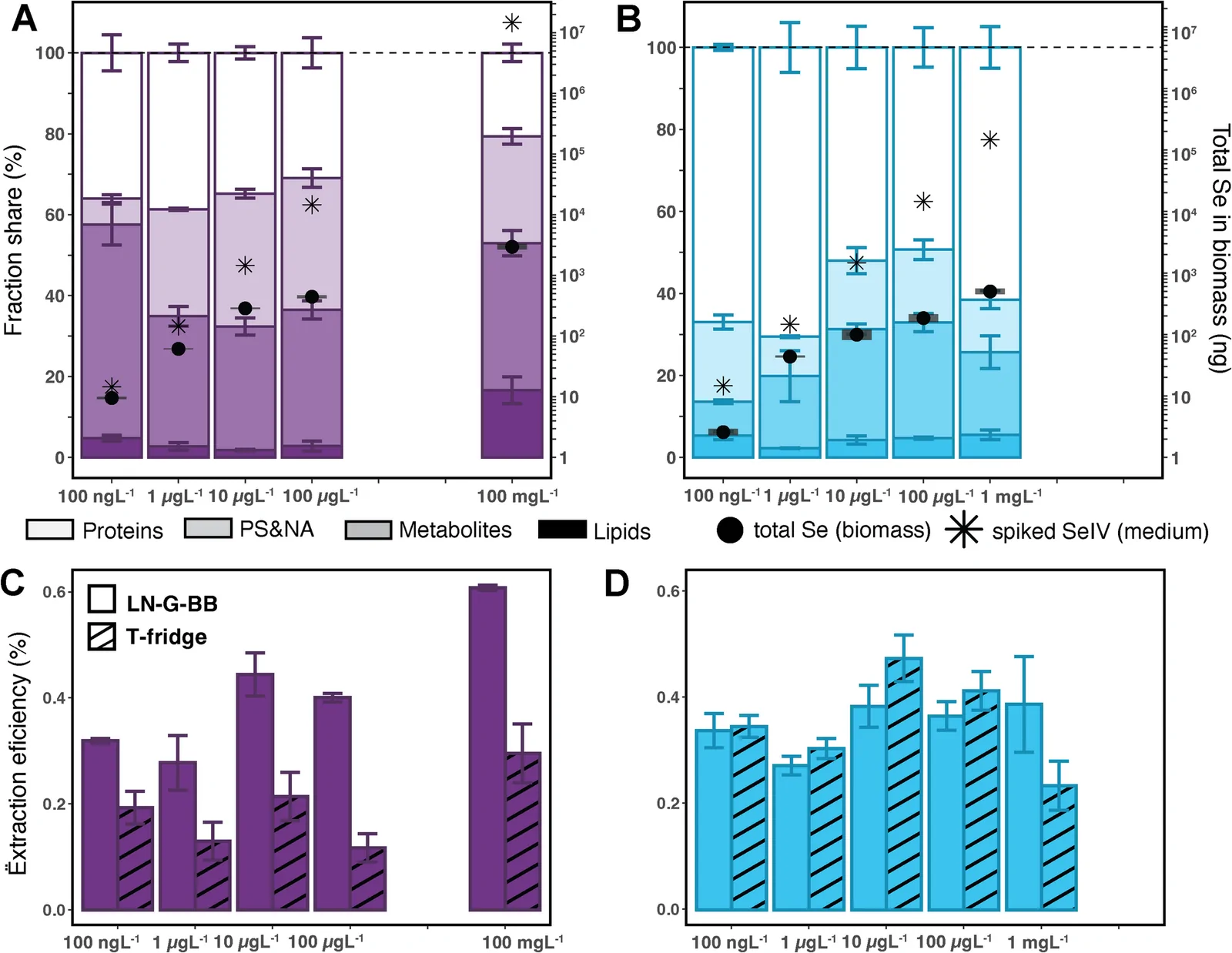
Se uptake, intracellular partitioning and extraction efficiencies vary with medium Se(IV) concentrations. **A** (*E. voratum*) and **B** (*G. huxleyi*): Barplots show the relative distribution of Se between intracellular fractions in percent. Black points show the sum of all intracellular fractions in ng for the whole algal biomass growing per culture, black stars indicate the total amounts of Se(IV) available to the algae (ng), both log scaled. **C** (*E. voratum*) and **D** (*G. huxleyi*): Extraction efficiencies calculated by the ratio of total Se found

Expectedly, in both algae, increasing medium [Se(IV)] resulted in higher total intracellular Se contents, from 1.0 ± 0.1 to 101 ± 15 µM in *G. huxleyi*, and from 5.2 ± 0.2 to 193 ± 11 µM in *E. voratum* for medium [Se(IV)] of 0.1 to 100 µg Se L^−1^ (note: intracellular [Se] is reported in molar concentrations here to account for differences in cell size between the two phytoplankton species; black circles in Fig. 7A & B show results in total ng for easier comparison with medium Se loads, i.e., black stars in the same figure). However, the efficiency of Se assimilation decreased in the meantime as bioconcentration factors (i.e. [Se]_intracellular_/[Se]_medium_) declined exponentially from 4144 to 12.4 for *E. voratum* and from 825 to 28.6 for *G. huxleyi* when medium [Se(IV)] increased from 0.1 to 100 µg Se L^−1^, respectively. Changes in the intracellular Se distribution were also observed with increasing medium [Se(IV)] as determined by sequential extraction (bars in Fig. 7A & B, see Fig. S14 for analytical mass balances). In higher Se biomass, the share of Se proteins in *E. voratum* generally decreased together with a concomitant increase in the amount of Se in PS&NA. In the highest concentration tested (100 mg Se L^−1^), Se in lipids also increased, even though this fraction generally holds little Se. In the case of *G. huxleyi*, we observed no consistent trend in the share of proteinaceous Se across the concentration range tested but generally a lower percentage of total intracellular Se was associated with proteins at high [Se], going along with a relative increase in the metabolite associated fraction. We find that this varying quantitative importance of Se in algal proteins depending on the medium Se concentration also directly affected Se extractions for HPLC-ICP-MS/MS measurements. In sect. 3.1.2. we discussed how different extraction protocols compare in terms of total Se recovery from algal cells, and we found that using an acidic buffer (glycine) in combination with bead beating (BB) had higher Se extraction efficiencies than e.g. simply incubating a cell pellet in neutral Tris buffer. In the concentration experiments, we found that the relative difference between these extraction techniques was also dependent on the medium Se concentration algae were growing in, especially for *E. voratum* (Fig. 7C). Here, the difference between Tris and Glycine increased with medium [Se(IV)], likely reflecting a lower proportion of Se in Se-proteins as Tris liberates a higher fraction of HMW-Se (as discussed in Sect. 3.2.1) (see Fig. S15 for a correlation between extraction efficiency and non-proteinaceous Se).

In summary, increasing medium concentrations in both algae resulted in increasing cellular Se loads, however it was more evident from very low to low concentrations (0.1 to 1 µg Se L^−1^) compared to from intermediate to high concentrations (10 to 100 µg Se L^−1^). Both algae showed changes in intracellular Se partitioning between cellular fractions; however, these changes differed in direction and magnitude, suggesting differential responses of different phytoplankton species to increased medium Se levels.

#### Effects of Se Concentration on Intracellular LMW Se Speciation

**Fig. 8:**
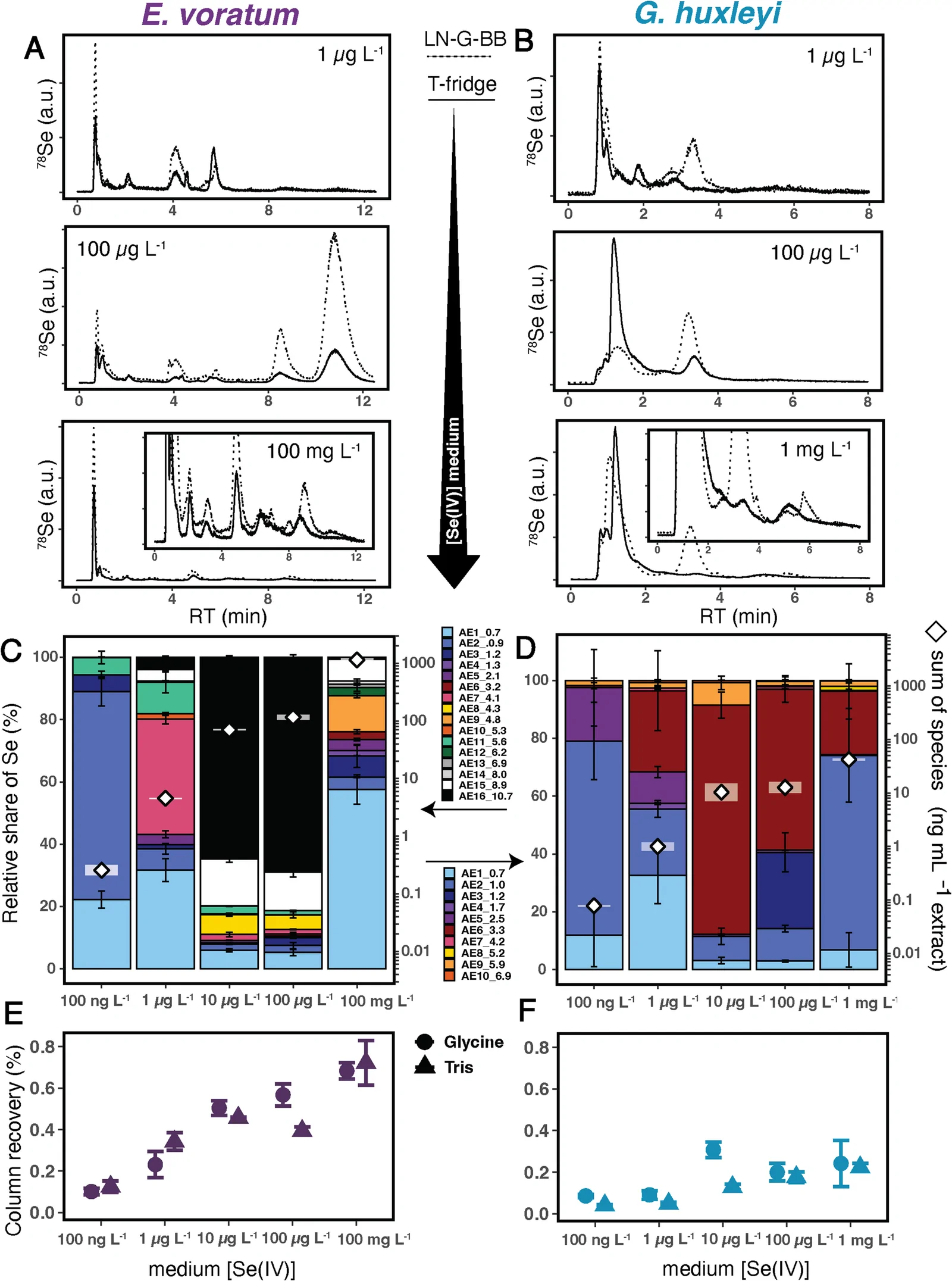
Anion exchange (AE) chromatography reveals changes in intracellular Se speciation in *E. voratum* (**A** & **C** & **E**) and *G. huxleyi* (**B** & **D** & **F**) grown under different media [Se(IV)]. **A** & **B** show chromatograms of representative glycine- (“LN-G-BB”; dotted line) and Tris-based (“T-fridge”; solid line) extracts. **C** & **D** show the relative proportion of peaks detected in these chromatograms (here shown for the “LN-G-BB” extraction technique) normalized to the sum of Se species (in ng mL−1) detected on the column which in turn is shown by white diamonds on the secondary y axis. Note that colors are not comparable between the panels **C** & **D**, as analyte retention times (RT) are not necessarily comparable between algal species because of different sample matrices. Note that integrations in such complex natural matrix samples can only be approximations as very frequently no baseline separations are reached, however they fulfill the objective of describing changes in relative speciation. **E** & **F** show column recoveries for the AE analysis, calculated as the sum of Se species eluting from the column relative to the total amount of Se present in the sample

Comparing Se speciation in *E. voratum* LN-G-BB extracts across the concentration gradient showed substantial changes between samples measured on the AE column (Fig. 8A & C and Fig. S16 for other concentrations and conditions). The transition from 1 µg Se L^− 1^ to 10 and 100 µg Se L^− 1^ was marked by the appearance and increase of two prominent, highly-retained peaks in *E. voratum* extracts (AE15 and AE16 at RT = 8.9 min and 10.7 min, respectively), which were more abundant in the LN-G-BB than in the T-fridge extracts. Further increasing medium [Se(IV)] to 100 mg Se L^− 1^ again strongly changed Se speciation, with AE15 and AE16 representing only a minor share of the measured species, but the appearance of additional, less-retained Se-containing peaks (AE6, AE9, AE11 at RT = 3.2 min, 4.8 min and 5.6 min, respectively) and a large amount of Se shifted to or near the column void (AE1, RT = 0.7 min).

As described in sect. 3.2.2, column resolving power of AE for *G. huxleyi* extracts is low. Nevertheless, we could still observe general shifts in AE Se speciation with increasing medium [Se(IV)] also for this algae (Fig. 8B & D). Furthermore, analyzing these extracts with the mixed-mode column demonstrated the formation of distinct species at high concentration (Fig. S16). These species were highly retained (RT = 8, 9, and 14 min) and could not be detected in samples grown below 10 µg Se L^− 1^, further supporting the finding that in both microalgae Se speciation changed considerably with medium Se concentration.

Lastly, we note that increasing medium Se(IV) concentrations in these experiments also affected total column recoveries in the AE HPLC-ICP-MS/MS analysis. Extracts from low medium [Se] *E. voratum* cultures – containing more Se in proteins and PS&NA – show dramatically lower AE column recoveries than those from high medium [Se] cultures (Fig. 8E). This effect was also observed, although less prominent for *G. huxleyi*, reflecting the less drastic changes to its intracellular Se distributions (Fig. 8F). Overall, these observations are consistent with conclusions from sect. 3.2.2.

## Summary and Conclusion

In this study, we provided a systematic assessment of how technical and methodological choices in an experiment investigating intracellular Se speciation in marine phytoplankton will affect the experimental results. We found that phytoplankton cells should be harvested by centrifugation and thoroughly washed with an isotonic, phosphate containing washing solution, in order to remove excess, extracellularly-bound inorganic Se from the cell pellet. SEC-ICP-MS/MS applied to Tris-SDS based algal extracts proved to be a valuable technique for quantifying both HMW and LMW Se species in phytoplankton extracts, and we were able to link these fractions to the intracellular Se distribution patterns that can be achieved by sequential extractions. When optimizing cell extractions for high extraction efficiencies but low releases of HMW Se species that might obstruct small-bore analytical HPLC columns, we achieved best results by quenching cells with liquid nitrogen followed by cell resuspension in an acidic buffer and the application of shear stress (cryogenic bead beating, sonication, or microwaving). The choice of the chromatographic column has a large impact on the experimental results: while cell extracts of *E. voratum* and *G. huxleyi* indicated similar Se speciation on the mixed-mode column, the same samples showed very different speciation results when analyzed using the anion-exchange column. Awareness of potentially very low column recoveries is key when dealing with such complex matrix samples, and we found this problem to be exacerbated when working with cells grown at close to natural medium [Se(IV)]. Enriching phytoplankton culture media with high amounts of Se had strong, quantifiable impacts on both the intracellular distribution of Se between different biochemical fractions, as well as Se speciation measurements in cell extracts. Importantly, such changes were already observed when deviating from environmental (low µg L^− 1^) Se concentrations. Additional changes in measured Se occurred when moving to toxic levels (mg L^− 1^).

Overall, the different analytical approaches presented in this study jointly highlight substantial differences between Se interactions with *E. voratum* and *G. huxleyi*. The dinoflagellate accumulated substantially more Se than the coccolithophore, and at the same time, was considerably less affected by Se toxicity. This latter observation aligns with the lower relative proportion of Se found in proteins, which are generally considered the most bioactive forms of Se in cells, i.e. may also be a source of Se-toxicity. The higher relative Se extraction efficiencies observed for *E. voratum* likely also reflect this lower proportion of protein-bound Se and a greater contribution of LMW compounds, even though an impact of differing cellular architecture cannot be excluded. Anion-exchange chromatography demonstrated the presence of a substantial pool of strongly anionic Se species in *E. voratum*, which increased in relative importance at higher medium Se concentrations. We speculate that these species might represent Se-sugars, anionic Se compounds for example also found in mammalian tissue under elevated Se supply [73], and present in urine likely serving Se excretion [74] or accumulating in mammalian tissues under elevated Se supply. Their formation could potentially contribute to the lower Se toxicity observed in *E. voratum* compared to *G. huxleyi*, which did not show comparable production of these compounds. Future research may extend these findings not only towards molecular characterization of Se-metabolic products formed under Se-toxicity in different algal species, but also by investigating whether the observed increased cellular Se uptake of *E. voratum* compared to *G. huxleyi* translates into stronger Se dependence under Se limitation. This could be assessed, for example, by determining Se half saturation constants for both species under varying environmental growth conditions.

The finding of a strongly altered Se-metabolism under high Se exposure, especially in *E. voratum*, underscores the unique potential of atomic mass spectrometry in marine phytoplankton Se research: while Se enrichments are useful for identifying individual Se-containing compounds by molecular mass spectrometry, it should be noted that compared to natural phytoplankton growing with little Se in the ocean, certain Se species may be over-represented, or not present at all. Combining both atomic and molecular mass spectrometry in experimental designs can broaden future research, enabling not only the identification of Se compounds from enriched biomass but also the verification of their environmental relevance.

## Supplementary Information

Below is the link to the electronic supplementary material.

Supplementary Material 1 (DOCX 2.60 MB) 

## Data Availability

Data will be made available upon request.
