# Supplementary material for: Exploring Intracellular Se Speciation in Marine Phytoplankton by LC-ICP-MS/MS: from Se Enrichments to Extraction and Chromatographic Techniques
Source: Biol Trace Elem Res. 2026 Apr 21;204(8):5767–88. doi: 10.1007/s12011-026-05069-7 (PMC13652914; doi:10.1007/s12011-026-05069-7)
Supplement: Supplementary file 1 [file 12011_2026_5069_MOESM1_ESM.docx]

**Supplementary Information to**

**Exploring intracellular Se speciation in marine phytoplankton by LC-ICP-MS/MS: from Se enrichments to extraction and chromatographic techniques**

Béziat, Pauline^a,b^ ; Bouchet, Sylvain^a,b,1^; Tolu, Julie^a,b^ ; Alcolombri, Uria^c^ ; Winkel, Lenny H.E.^a,b^

a -Institute of Biogeochemistry & Pollutant Dynamics, Dept. of Environmental Sciences, Swiss Federal Institute of Technology (ETHZ), Universitätstrasse 16, 8092, Zurich, Switzerland

b - Department Water Resources and Drinking Water, Swiss Federal Institute of Aquatic Science & Technology (Eawag), Überlandstrasse 133, 8600, Dübendorf, Switzerland

c -The Alexander Silberman Institute of Life Sciences, The Hebrew University of Jerusalem, Edmon J. Safra Campus – Givat Ram, Jerusalem 9190401, Israel

1 - present address: Federal Institute of Metrology (METAS), Lindenweg 50, 3003 Köniz, Switzerland

^*^ Corresponding author: [pauline.beziat@usys.ethz.ch](mailto:pauline.beziat@usys.ethz.ch); [sylvain.bouchet@usys.ethz.ch](mailto:sylvain.bouchet@usys.ethz.ch)

**(28 pages, 16 figures, 2 tables)**

**Table of contents**

[S1: Review on previous measurements of Se speciation in microorganisms	2](#_Toc217025585)

[S2: Se measurements by ICP-MS/MS	7](#_Toc217025586)

[S3: Effect of cell washing	11](#_Toc217025587)

[S4: SEC-ICP-MS/MS analysis	12](#_Toc217025588)

[S5: Choice of chromatography & impact of extraction techniques on speciation	18](#_Toc217025589)

[S6: Impact of medium Se concentration	24](#_Toc217025590)

[S7. References	27](#_Toc217025591)

# S1: Review on previous measurements of Se speciation in microorganisms

**Table S1 : Previous studies on Se speciation in algae (endo and exometabolome), as well as intracellular Se analyses in bacteria**

| **EXOMETABOLOME ALGAE** | | | | | | | | |
| --- | --- | --- | --- | --- | --- | --- | --- | --- |
| **Publication** | **Species** | **Medium** | **Se concentration** | **Cell treatment** | **Chromatographic separation (if used)** | **Se detection method** | **Speciation found** | **Column recovery or extraction efficiency** |
| LeBlanc et al., 2012 [1] | *Chlorella vulgaris, Chlorella kesslerii, Scenedesmus obliquus ,Synechoccus leopoliensis*  *Natural river with blue/-green algae bloon* | Bold’s basal medium | Bold’s basal medium | Medium only | AEC Dionex ICS-90 with Dionex IonPac AS14A 3x15mm | ICP-DRC-MS and ES-MS/MS | SeCN- | - |
| LeBlanc et al., 2016 [2] | *Chlorella vulgaris, Chlorella kesslerii, Scenedesmus obliquus* |  | 10 or 25 µg L^-1^ as Se(VI) | Medium only | AEC Dionex AS16 (250mm 10/32) | ICP-DRC-MS and ES-MS/MS | SeMet, SeOMet **by ES-MS/MS**, MeSeCys , MeSe(IV), SeCN- and cyclic SeOMet **by AEC RT matching** | - |
| Simmons & Wallschläger, 2011 [3] | *Chlorella vulgaris* | 10% Bolds Basal medium | 10 µg L^-1^ as Se(VI), Se(IV) or SeCN- | Medium only | AEC Dionex AS16 (4mm 10/32) | ICP-DRC-MS | SeCN-, Se(IV) and Se(VI) by **AEC RT matching** | - |
| Simmons and Emery, 2011 [4] | *Chlorella vulgaris* | 10% Bolds Basal medium | 0.5, 2.7, 5.5 µg L^-1^ Se(VI) | Medium only | AEC Dionex AS16 (4mm 10/32) | ICP-DRC-MS | SeCN-, Se(IV) and Se(VI) by **AEC RT matching** | - |
| Besser et al., 1994 [5] | *Chlamydomonas rheinhardtii* |  | Algae grown at 100 µg or 1000 µg L^-1^ Se (3:1 SeVI to SeIV) | Medium only | Combination of charcoal, Cu-chelex and sephadex | Gamma counter | Grouping into inorganic, Se-amino and non-amino fractions | not applicable |
| **ENDOMETABOLOME** | | | | | | | | |
| Zdziebłowska et al., 2024 [6] | *Arthrospira platensis* (freshwater cyanobacterium) | Ultra-pure water based medium | 10 mg L^-1^ Se(IV) | Cells were dried and ground, extracted by vortexing at 37 °C for 45 min with 2 mL of (i) 2 % SDS, (ii) 30mM Tris-HCl, (iii) 5 % pectinase (iv) 5 % cellulase or (vi) 5 % hemicellulase | SEC Superdex (200 10/300 GL); SeQuant ZIC-HILIC (150 x 2.1 x 3.5 µM) | ICP-MS/MS and ESI-MS/MS with JetStream technology | Various compounds including Semet, selenoadenosine, selenohomolantionine | Extraction efficiency reported as 89 – 103 % |
| Guo et al., 2024 [7] | *Chlorella kessleri (freshwater Chlorophyceae, green)* | mGB-11 | 8 mg L^-1^ Se(IV) | Centrifugation, washing with MilliQ, lyophilization, extraction according to Wang et al., 2023 (see below) | Wang et al., 2023 | Wang et al., 2023 | SeCys, SeMet, MeSeCys,  based on AEC RT matching (no chromatogram shown) (proteinaceous) | - |
| Wang et al., 2023 [8] | *Tribonema minus*  *(yellow-green freshwater, SAR)* | Modified blue-green medium | 0.5 – 8 mg L^-1^ Se(IV) | According to Stadlober at al., 2001: lyophilization, pronase E and ultrasonication at 37°C for 1h. | PRP-X-100 (250mm x 4.1mm, 10µm) | ICP-MS | SeCys, SeMet, MeSeCys, based on AEC RT matching (no chromatogram shown) (proteinaceous) | Yields reported as inorganic vs. organic |
| Mylenko et al., 2020 [9] | *Chlorella vulgaris (freshwater Chlorophyceae, green)* | Modified inorganic medium | 0.5, 1, 4 or 8 mg Se(IV) per g culture dry weight, twice a day | Methods according to Vu et al., 2019 | See below | See below | SeCys, SeMet, MeSeCys | Reported total algl concentrations and concentrations of SeCys, SeMet, MeSeCys |
| Vu et al., 2019 [10] | *Chlorella vulgaris R117 (green)* | Modified inorganic medium | 2.25 – 4.5 mg L^-1^ Se(IV) | Centrifugation, washing with water, disruption using a high-pressure homogenizer, spray drying. Protein hydrolysis with 3M methane sulfonic acid containing mercaptoethanol (24h, 95°C). Derivatisation according to [11] and [12]. | PRP-X-100 (4.1 x 150mm, 10µm) | GC-APCI-HRMS or HPLC-ICP-MS | SeCys, SeMet, MeSeCys | Total cellular Se not determined |
| Vriens et al., 2016 [13] | *Chlamydomonas rheinhardtii* | Talaquil | 2.4 or 6 mg L^-1^ Se(VI) or Se(IV) | Filtration, rinsing with 25mM PO4 buffer pH 7.5 | - | XANES and EXAFS | Mainly unknown organic Se species except for when grown at 6mg L-1 Se(IV): mainly Se[0] | Not applicable |
| Da Silva et al., 2013 [14] | *SRM plankton BCR-414*  *(freshwater, lyophilized – 95% D.magna (=zooplankton))* | NA | NA | Extraction in MilliQ by sonication for 2hours, lophilisation | PRP-X-100 (4.6 x 250mm, 7µm) | DRC-e ICP-MS | SeCys2, SeIV, SeVI, SeMet (< LOD) | Extraction efficiency 29%, recovery 91% |
| Gómez-Jacinto et al. 2012 [15] | *Chlorella sorokiniana*  *(freshwater Chlorophyceae, green)* | Freshwater, M-8 medium | 50 mg L^-1^ Se(VI) | Centrifugation, cell breaking with LN, addition of protease XIV and sonication for 2 min. | Phenomenex Luna C18 (250mm x 4.6mm, 5µM)  Astec Chirobiotic T column (250 x 4.6mm, 5µm) | ICP-MS/MS | In protease XIV extracts: SeCys2, MeSeCys, SeMet, Se(VI), **based on C18 RT matching** (proteinaceous) | - |
| Gómez-Jacinto et al. 2012 [16] | *Chlorella sorokiniana* *(freshwater Chlorophyceae, green)* | Freshwater, M-8 medium | 50 mg L^-1^ Se(VI) | Sequential extraction according to Casens et al., 2002:  (i) agitation in hot water  (ii) 4% Driselase in 30mM Tris-HCl (pH 7) with 1mM PMSF  (iii) 30mM Tris-HCl with 4% SDS  (iv) phosphate buffer (pH 7.5) at 37°C with protease XIV | SEC Superdex-75 (10mm x 300mm, 13µM), sep. range 3-70kDa;  Phenomenex Luna C18 (250mm x 4.6mm, 5µM) | ICP-MS/MS | In protease XIV extracts: SeCys2, MeSeCys, SeMet, Se(VI), SeCys, **based on C18 RT matching** (proteinaceous) | - |
| Umysová et al., 2009 [17] | *Scenedesmus quadricauda*  *(freshwater Chlorophyceae, green)* | Liquid mineral medium | 5 - 400 mg L^-1^ Se(IV) or Se(VI) | According to Wrobel et al., 2003: mixing biomass with 4M methanesulfonic acid and 2-mercaptoethanol, 16h reflux | PRP-X-100 (4.6 x 150 mm) | ICP-MS | SeMet by AEC RT matching (proteinaceous) | reporting %SeMet of total cellular Se (8.3 – 40.8%) |
| Obata et al. 2004 [18] | *Emiliana huxleyi*  *(marine SAR, red)* | Marine, ASW Erd-Schreiber’s medium | 79 µg L^-1^ as ^75^Se(IV) | Centrifugations, washing with artificial seawater, extraction with CHCl_3_-MeOH, KCl and TCA, preconcentration | *-* | Thin Layer Chromatography | *5 unknown LMW Se,* no Se(IV), SeMet, SeCys visible | *-* |
| Neumann et al. 2003 [19] | *Chlorella sp.*  *(freshwater Chlorophyceae, green)* | Freshwate, DI + nutrient solutions | 1.58 mg L^-1^ | Centrifugatiom, washing in disitilled water, suspension in glycerin | - | XAS | Se(IV), Se(VI), SeCys_2_ and something with a C-Se-C moiety | Not applicable |
| Fan et al., 2002 [20] | *Natural microphytes* | *filtered from Se-lades agricultural evaporation ponds* | 5.1 – 13.5 µg L^-1^ total dissolved Se | Centrifugation, washing with isotonic solution, lyoph-ilization, ball milling, boiling in 62.5mM Tris-HCl, 0.2% SDS, 5% mercapto-ethanol, dialysis to remove LMW compounds. Digestion of protein pellet for Se-Met analysis in HCl, phenol and thioacetic acid | - | GC-MS | Proteinaceous SeMet, low in free SeMet | Extraction efficiencies in Tris-SDS 43-89%, 11-64% present as proteinceous |
| Larsen et al. 2001 [21]q | *Chlorella sp. from agricultural evaporation pond*  *(euryhaline Chlorophyceae, green)* | Euryhaline, “Seawater medium” + f/2 | 100 mg L^-1^ Se(IV) | Centrifugation, Washing, Lyophilisation, pulverization with Teflon Ball Mill, NaOH treatment, extraction with TCA, again freeze-drying | - | Cation and Anion Exchange HPLC-ICP-MS, ESI-MS | DMSeP, SeCys_2_ or SeOMet, AllSeCys and SeEt | - |
| Fan et al. 1997 [22] | *Chlorella sp.*  *(euryhaline Chlorophyceae, green)* | Euryhaline, natural seawater + f/2 | 100 mg L^-1^ | No information on cell isolation and washing; freeze-drying and pulveri-zation using a Teflon ball mill, extraction with TCA or PCA, preconcentration (freeze-drying), derivatization and sonication | - | GC-MS and multi-nuclear NMR | Trace amounts of SeMet, unkown compound, no SeCys, Se(IV) or Se(VI) | - |
| Bottino et al. 1984 [23] | A: *Dunaliella tertiolecta* B: *Chlorella sp.*  *(both marine Chlorophyceae, green)*  C: *Porphyridum cruentum*  (no speciation for P.cruentum) | Marine, “Mueller’s Artificial Seawater Medium” | 100 mg L^-1^ as ^75^Se(IV) | Centrifugation, washing with Medium, treatment with TCA and tissue grinder, CHCl_3_-MeOH, 80% hot ethanol, evaporation, protein extraction with pronase | Ion-Exchange Chromatography (Dowex 50W-X8-400) | AAS  Standards: SeEt, SeCysta, SeCys, SeCys_2_ | A: *Selenocysteic Acid*^[1]^, *MeSeCys*^[2]^, SeCys, SeCys2, SeMet, *MeSeMet*^[3]^  B: *Selenocysteic Aci*d^1^, *MeSeCy*^2^, SeCys, SeCys2, SeMet | Provide biochemical fractionation (hence also extraction efficiencies) |
| Wrench et al. 1978 [24] | A: *Tetraselmis tetrathele* B: *Dunaliella minuta*  *(both marine Chlorophyceae, green)* | Marine, sterile culture media + nutrients | 5µCi ^75^Se(IV) | Centrifugation, extraction with CHCl_3_-MeOH, vacuum-distillation to dryness, protein extraction with pronase | *-* | Technicon amino acid autoanalyzer,  Identification only based on RT of S compounds | *A & B: Selenocysteic Acid^1^*,  *SeMet-O, very tentatively MeSeCys, SeMet, SeCys, Se(IV)* | *-* |
| **ENDOMETABOLOME OTHER MICROORGANSIMS** | | | | | | | | |
| **Publication** | **Species** | **Medium** | **Se concentration** | **Cell treatment** | **Chromatographic separation (if used)** | **Se detection method** | **Speciation found** | **Column recovery or extraction efficiency** |
| Kayrouz et al., 2024 [25] | **Bacteria:**  M.vinifirmus DSM17747  H.utahensis DSM3051 | Bacto marine broth Difco2216 | 4 mg L^-1^ Se(IV) | Centrifugation, resuspension in MeCN with 10mM mBBr for derivatization, sonication for 30min at room temperature | RP: SyngeriFusion Phenomenex 100x4.6mm, 4µm | HPLC-MS (Q-tof) | ovoselenol | *-* |
| Kayrouz et al., 2022 [26] | **Bacteria:**  Amycolatopsis palatopharyngis DSM 444832; Variovorax paradoxus DSM 30034 | Tryptic soy broth, nutrient broth | 8 mg L^-1^ Se(IV) | Centrifugation, resuspension in MilliQ, hot-water extraction by heating to 95°C for 15 min | RP: SyngeriFusion Phenomenex 100x4.6mm, 4µm | HPLC-MS (Q-tof) | selenoneine | *-* |
| Michalke et al., 2001 [27] | **Unknown, non-pathogenic bacterial sample** | Unknown | unknown | Protease digestion in Tris-HCl 10mM, pH 7.3 for 24h in a water bath, dilution 1:1.5 before measurement | AEC: Dionex AS11 | HPLC-ICP-MS | SeU, SeMet, SeIV, SeCys2, SeVI | Column recovery 98.7%, extraction efficiency of 25% |

# S2: Se measurements by ICP-MS/MS

**Table S2 (HPLC)- ICP-MS/MS conditions employed for the determination of total Se**

| **ICP-MS/MS** | **Agilent 8800** | |
| --- | --- | --- |
| RF power | 1500 W | |
| Nebulizer | Conikal (HPLC), Micromist (total analysis) | |
| Nebulizer gas | Tuned daily (1.06 – 1.15) | |
| CRC gas flows | H_2_ mode: 5ml min^-1^ H_2_  O_2_ mode: 2 ml min^-1^ H_2_, 30% O_2_ | |
| Se Isotopes (mass) / dwell times (sec) | HPLC measurements:   0.1 sec for Se 77, 78, 80 in H_2_ mode   Total measurements:  0.5 sec for Se 76, 77, 78, 80, 82 in O_2_ and H_2_ mode | |
| **HPLC settings** | | |
| **Column** | **Mobile phases** | **Gradient** |
| IonospherC | A : 0.8mM pyridine formate, pH 3, 1% MeOH  B : 8mM pyridine formate, pH 3.2, 1% MeOH | 0 – 7 min : 100% A  7 – 7.5min : 100% B  7.5 – 37min : 100% B  37 – 38 min : 100% A  38 – 47 min : 100% A |
| PRP-X-100 | A : 40mM ammonium citrate pH 5.2, 2% MeOH  B : MilliQ | 0 min : 4 % A  4 min : 10% A  4.5 min – 10min : 25% A  11 – 12.5 min : 4% A |

**2.1. Microwave assisted digestions**

In preparation of the total Se measurements by ICP-MS/MS, samples (liquid extracts as well as solid cell residues) were acid digested using the following program: 10 min ramp 40 – 100 °C, 5 min 100°C, 15 min ramp to 220 °, 10 min hold and max pressure 120 bars. All samples were diluted to a final HNO_3_ concentration of 10 % (v/v) for measurement by ICP-MS/MS.

**2.2. Correction of instrumental drift and QC for total ICP-MS/MS measurements**

We assessed the use of Y and Rh (introduced together in a 1% HNO3 solution continuously infused using a T piece) for correcting the instrumental sensitivity drift during every analytical batch by considering 1) the recovery of a 5 µg L^-1^ QC standard (run every 10 samples) and 2) the recovery of our digestion SRM, IAEA-392. On the basis of these results, in all except for one batch we chose the ISTD Y for correcting matrix induced changed in Se sensitivity.

We recorded all isotopes 76, 77, 78, 80 and 82 Se in both H_2_ and O_2_ mode. For every batch and isotope, all measurements were excluded that were below the LOD determined as the average counts across all blanks plus three times the standard deviation. In H_2_ mode, ^82^Se, ^80^Se, ^78^Se and ^77^Se were corrected for the hydration of ^81^Br, ^79^Br, ^77^Se and ^76^Se, respectively. Average hydridation factors across all analytical batches were 0.51 ± 0.19 % for fSe and 0.61 ± 0.27% for fBr. As the samples in this study consist in high and variable matrix samples with high salt content, careful monitoring and assessment of isotopes is required. In phytoplankton digests, 77, 80 and 82 (H_2_ mode) usually provided the most robust results, only in PS&NA and KCl (i.e. “metabolites”) samples, 77 was not usable (highly interfered) and in particularly low Se samples the isotope 82 had higher errors and had to be excluded. Across analytical batches, the considered isotopes in algae samples had average agreements of 6.3 ± 3.66 %.

The IAEA-392 SRM “Trace, minor and major elements in algae” does not have a certified Se concentration, but a suggested GeoRem concentration of 0.19 – 0.11 µg g^-1^ [28]. While the first bottle of IAEA-392 we used yielded average Se concentrations of 0.09 ± 0.04 µg Se g^-1^ (Lucie Sauzéat, pers. communication), the material appears to be heterogeneous as the second bottle of the same SRM in our hands showed average concentrations of 37 ± 1 ng g-1 (both determined via acid digestion and total Se measurement by ICP-MS/MS). We henceforth adapted 37 ± 0.1 ng g-1 as the true Se concentration of this SRM for this study. For total Se digestions and measurements of the samples presented in this work, one aliquot of IAEA-392 (50 – 60 mg) was digested and measured for every 13 experimental samples. In these digestates, we found that isotopes 76 was always strongly interfered in H2 mode, however interferences were variable, for isotopes 77 and 78 interferences occurred only in select samples. Using 80 and 82 Se across all analytical batches, we found an average SRM recovery of 90.6 ± 10.7 % (n=31). In some cases, the liquid SRM NIST1643f “Trace elements in water” was used as an additional verification. In this case we found average Se recoveries of 98.19 ± 2.3 % (1:100 diluted, all isotopes, O_2_ and H_2_ mode).

**Figure S1:** Impact of the MeOH content on Se standards (prepared in milliQ water without buffer) elution for the Hypercarb column.

# S2: Effect of cell washing

**Figure S2:** Concentrations of Se species in cells of *E. voratum* (A & B) and *G. huxleyi* (C & D) extracted in Tris-HCl after quenching with LN and harvesting either by centrifugation (A & C) or filtration onto GF/F filters (B & D). Cells were either washed with centrifugation supernatant (i.e. cell-free culture medium, “no wash”), with a 0.6M NaCl solution containing 2.4 mM NaHCO_3_ (i.e. 1 times washing with this solution denoted as 1 x) or with the same solution additionally containing 25 mM PO_4_. Bars show the measurement of unknown organic Se species in extracts by the mixed mode chromatography (e.g. H3 = 3^rd^ peak on the Hypercarb column with retention time of 2.2 min), points show the concentrations of species eluting at 1.8 min which matches in RT with Se(IV), the Se form supplied to the algal medium.

# S4: SEC-ICP-MS/MS analysis

We used 2 columns connected in series (Superdex Increase 75 and Superdex Peptide) to obtain a separation of compounds up to ~70 kDa. The capacity of these columns to separate macromolecules from Se-containing LMW standard was investigated using a set of analytical standards (see below**, Fig. SI 3** and **SI 4**).

All the tested proteins including the smallest one, aprotinine (6.5 kDa, RT = 27.1 min), were well-resolved from the earliest eluting LMW Se standard (Se(VI), RT = 28.0 min). However, two relatively small macromolecules, i.e. polystyrene sulfonic acid (1.1 kDa RT = 28.7) and vitamin B12 (RT = 34.6) eluted in the retention time window of the LMW Se standards used in this study (RT = 28.0 – 50.3 min). This window is quite wide as the selected column exhibits not only a size exclusion but also a cation exchange mechanism, inducing retention of cationic Se species such as TMSe^+^ or MeSeMet^+^ as well as repulsions of anionic species, such as Se oxyanions. Due to this ionic exchange behavior of the column, the size detection is limited to around 5–6 kDa based on the RT window defined above. While the wider range of RT for LMW compounds extends analysis times, it would be advantageous for application to molecular mass spectrometry as co-elution and ionization suppression is limited.

**Figure S3:** Elution of the various low and high molecular weight standards tested with the Superdex peptide column. **A:** LMW Se standards (trace shows Se80, measured in H_2_ mode), blue: inorganic Se species (note – SeIV does not elute), dark red: seleninic acids, green: selenides, cyan: diselenide, purple: selenonium, * indicates unknown contaminants in the synthesized DMSeP and MeSeMet standards. Se measured **B:** protein mix standards as recorded by their sulfur signal (S32 – 48, O2 mode) (sizes are in Da).

**Figure S4:** Molecular weight vs. retention time of different types of standards measured on the Superdex peptide column: proteins (red squares), polystyrene sulfonic acids (green diamonds), vitamin B12, a coordination complex (grey circle) and low molecular weight Se standards (Se-amino acids, inorganic Se etc., see Fig SI 2A, orange triangles). The black and grey background illustrates what sizes have been classified as LMW and HMW in the analysis of algal Se compounds (see Fig., 2 E & F). HMW Se species of *S.voratum* by RT are larger than 158 kDa, in the case of *G.huxleyi* larger than 44kDa. LMW Se species fall in the window of Se-amino acids but in the case of *S.voratum* also overlap with protein size standards up to 17 kDa, in the case of *G.huxleyi* with protein size standards up to 6.5 kDa. The white dotted line marks the RT of the smallest protein size standard.

**Figure S5:** SEC-ICP-MS/MS chromatograms of *E. voratum* extracts: LNT-T-Sonic (solid line) and LN-T-Sonic-SDS (dotted line).

**Figure S6:** SEC-ICP-MS/MS chromatograms of *G. huxleyi* extracts: LN-T-Sonic (solid line) and LN-T-Sonic-SDS (dotted line).

**Figure S7:** SEC-ICP-MS/MS chromatograms of *E. voratum* (A-C) and *G. huxleyi* (D-F) extracts. Solid lines show the average chromatogram of three extraction replicates, the shaded area shows the respective standard deviation. Plots show the effects on “HMW “and “LMW” Se species of changing the extraction buffer or matrix from the neutral Tris-HCl to the acidic Glycine (A & D), from aqueous Tris-HCl to organic MeOH (B & E) and from Tris-HCl to Tris-HCl with driselase enzymatic mix (C & F).

# S5: Choice of chromatography & impact of extraction technique on speciation

**5.1. Standard elution**

Of the 9 LMW Se standards used in this study, 7 standards were resolvable on the mixed-mode column within a run time of 8 min (SeEt at 7.8 min, not shown in **Fig. 3**), 6 standards on the anion-exchange column within 11 min, and 8 standards on the cation-exchange column within 40 min. Out of the 4 columns tested in this study, the cation-exchange is the only one that can reliably separate the putative algal metabolites DMSeP and MeSeMet from the other standards. All columns are affected by matrix effects (**Fig. S8**) but they are stronger on the cation-exchange column than others, where especially the presence of glycine buffer in the sample strongly affects elution of standards. Hence, while the use of acidic glycine for extraction is advantageous in terms of producing extracts with high concentrations of LMW Se but minimizing the accumulation of HMW Se, this buffer would not be the appropriate choice if the cation-exchange column is to be used as the main tool for extract analysis. For the anion-exchange and the mixed-mode columns the presence of glycine is less problematic; only the separation between MeSeCys and Se(VI) was affected on the latter. On the other hand, Tris buffer affected the separation between MeSeCys and Se(IV) on the anion-exchange column.

**Figure S8:** Example chromatograms (zoom on the first 3.5 min of the 35 min run) of an algal extract (purple: *E. voratum*, turquoise: *G. huxleyi*) prepared by LN-T-micro and analysed by cation exchange chromatography (Ionospher-C column). The grey trace shows the elution of the first two standards, Se(IV) (very close to void) and MeSeCys (RT about 2.25 min).

**Figure S9:** Effect of buffer presence in the algae extract on the elution of Se standards from the cation-exchange (Ionospher), mixed-mode (Hypercarb) and anion-exchange (PRP-X-100) columns. No standard trace in MilliQ is available for the same day the standards were tested in buffer for the MM column, for a typical standard elution in MilliQ on the mixed-mode (different analysis day) refer to Fig 3 A. Standards: 1 – TMSe, 2 – SeIV, 3 – MeSeCys, 4 – SeVI, 5 – SeMet, 6 – SeCys2, 7 – SeEt, 8 – DMSeP, 9 – MeSeMet. Not depicted: SeEt (7) on AE elutes shortly after SeVI (4) and after 6 (SeCys2) on M.

**Figure S10:** Mixed-mode (MM), cation-exchange (CE) and anion-exchange (AE) column recoveries vs the % of HMW Se species (as assessed by SEC) for both aqueous and methanolic extracts of both algal species.

However, that correlation in our case only materialized for aqueous *G. huxleyi* extracts (dark blue), not for the methanolic or any of the *E. voratum* (purple) ones.

**Figure S11: Effect of sample extraction technique on Se speciation in *E. voratum* (A & B) and *G. huxleyi (*C & D) extracts as measured by PRP-X-100, anion exchange**. **A and C:** Example chromatograms illustrating specific changes in chromatogram shape with different treatments. Solid lines show the average of three chromatograms with the shaded areas showing chromatogram standard deviations (extraction replicates).

**Figure S12:** Impact of sonication time on the abundance of peak H8 (RT in this case 6 min). Sample is an *E. voratum* extract (grown with 100 µg L^-1^ Se), measured by the mixed-mode column.

**Figure S13:** Mixed mode **c**hromatograms of first and second extracts of *E. voratum* extracted either by sonication for three minutes (A) or six minutes (B). Re-extraction (faint lines) of previously extracted phytoplankton pellets (solid lines) shows only peak MM-2 (RT 1.8, co-eluting with Se(IV)) and no other organic Se species. Note that this experiment was performed without PO_4_ in the washing solution, hence we expect the presence of excess Se(IV) as part of MM-2.

# S6: Impact of medium Se concentration

 **Fig S14:** **Closed** **mass balances of *E. voratum* (A) and *G. huxleyi* (B) extractions of the concentration experiment**. Every culture was split into 3 equal parts, centrifuged, washed and either extracted for measuring Se speciation by HPLC-ICP-MS/MS (using LN-G-BB (“Gly”) or T-fridge (“Tris”)) or intracellular Se distribution into biochemical fractions (metabolites, proteins, PS&NA and lipids). All samples were acid-digested and total Se was measured in digestates using ICP-MS/MS; for Tris and glycine extracts Se was measurements both in the extract (i.e. supernatant, SN) as well as in the residual cell pellet (i.e. solid residue (SR)). Small numbers above bars show the mean of sums (± 1 s.d.) of all fractions in ng per biomass pellet. Bars and error bars represent averages and standard deviations, respectively, of measurements between biological triplicates. Note changes in y-axis scaling between samples.

**Fig. S15:** **Se extraction efficiencies correlate with the fraction of non-proteinaceous Se in *E. voratum* (A) and *G. huxleyi* (B) across a concentration gradient.** Lighter points show extraction efficiencies for the glycine (LN-G-BB) extraction protocol, darker points represent those from the Tris (T-fridge) extraction protocol.

**Fig. S16: Chromatograms of *E. voratum* and *G. huxleyi* Tris-based extracts from cultures grown at increasing medium Se(IV) concentrations, analyzed by anion exchange (AE, top) and mixed mode (MM, bottom) chromatography.** All data presented here is detected as ^78^Se (H_2_ mode), the chromatograms from 100 ng Se L^-1^ cultures are presented as 5-point running averages. Note y-axis scaling factors as given in the respective panels.

# S7. References

[1]	K.L. LeBlanc, M.S. Smith, D. Wallschläger, Production and release of selenocyanate by different green freshwater algae in environmental and laboratory samples, Environ. Sci. Technol. 46 (2012) 5867–5875. https://doi.org/10.1021/es203904e.

[2]	K.L. Leblanc, D. Wallschläger, Production and Release of Selenomethionine and Related Organic Selenium Species by Microorganisms in Natural and Industrial Waters, Environ. Sci. Technol. 50 (2016) 6164–6171. https://doi.org/10.1021/acs.est.5b05315.

[3]	D.B.D. Simmons, D. Wallschläger, Release of Reduced Inorganic Selenium Species into Waters by the Green Fresh Water Algae Chlorella vulgaris, Environ. Sci. Technol. 45 (2011) 2165–2171. https://doi.org/10.1021/es103337p.

[4]	D.B.D. Simmons, R.J.N. Emery, Phytochelatin induction by selenate in Chlorella vulgaris, and regulation of effect by sulfate levels, ET&C 30 (2011) 469–476. https://doi.org/10.1002/etc.392.

[5]	J. Besser, J. Huckins, R. Clark, Seperation of Selenium Species Released from Se-Exposed Algae, Chemosphere 29 (1994) 771–780. https://doi.org/10.1016/0045-6535(94)90045-0.

[6]	S. Zdziebłowska, J. Zajda, L. Ruzik, Microalgae enriched in selenium as a good source of micronutrients, Food Bioscience 59 (2024) 103908. https://doi.org/10.1016/j.fbio.2024.103908.

[7]	Y. Guo, Y. Cao, R. Yang, F. Wang, N. Zhang, S. Li, Z. Zhu, Effects of three exogenous phytohormones on the growth, selenium absorption, and metabolism of the microalga Parachlorella kessleri under sodium selenite stress, Algal Res. 79 (2024) 103477. https://doi.org/10.1016/j.algal.2024.103477.

[8]	F. Wang, Y. Li, R. Yang, N. Zhang, S. Li, Z. Zhu, Effects of sodium selenite on the growth, biochemical composition and selenium biotransformation of the filamentous microalga Tribonema minus, Bioresour. Technol. 384 (2023) 129313. https://doi.org/10.1016/j.biortech.2023.129313.

[9]	M. Mylenko, D.L. Vu, J. Kuta, K. Ranglová, D. Kubáč, G. Lakatos, T. Grivalský, M.P. Caporgno, J.A. da Câmara Manoel, J. Kopecký, J. Masojídek, P. Hrouzek, Selenium Incorporation to Amino Acids in Chlorella Cultures Grown in Phototrophic and Heterotrophic Regimes, J. Agric. Food Chem. 68 (2020) 1654–1665. https://doi.org/10.1021/acs.jafc.9b06196.

[10]	D.L. Vu, K. Saurav, M. Mylenko, K. Ranglová, J. Kuta, D. Ewe, J. Masojídek, P. Hrouzek, In vitro bioaccessibility of selenoamino acids from selenium (Se)-enriched Chlorella vulgaris biomass in comparison to selenized yeast; a Se-enriched food supplement; and Se-rich foods, Food Chemistry 279 (2019) 12–19. https://doi.org/10.1016/j.foodchem.2018.12.004.

[11]	D.L. Vu, K. Ranglová, J. Hájek, P. Hrouzek, Quantification of methionine and selenomethionine in biological samples using multiple reaction monitoring high performance liquid chromatography tandem mass spectrometry (MRM-HPLC-MS/MS), Journal of Chromatography B 1084 (2018) 36–44. https://doi.org/10.1016/j.jchromb.2018.03.012.

[12]	P. Hušek, Z. Švagera, D. Hanzlíková, I. Karlínová, L. Řimnáčová, H. Zahradníčková, P. Šimek, GC-MS Metabolomic Profiling of Protic Metabolites Following Heptafluorobutyl Chloroformate Mediated Dispersive Liquid Microextraction Sample Preparation Protocol, in: G.A. Theodoridis, H.G. Gika, I.D. Wilson (Eds.), Metabolic Profiling, Springer New York, New York, NY, 2018: pp. 159–181. https://doi.org/10.1007/978-1-4939-7643-0_11.

[13]	B. Vriens, R. Behra, A. Voegelin, A. Zupanic, L.H.E. Winkel, Selenium Uptake and Methylation by the Microalga Chlamydomonas reinhardtii, Environ. Sci. Technol. 50 (2016) 711–720. https://doi.org/10.1021/acs.est.5b04169.

[14]	E.G. Da Silva, L.R.V. Mataveli, M.A. Zezzi Arruda, Speciation analysis of selenium in plankton, Brazil nut and human urine samples by HPLC-ICP-MS, Talanta 110 (2013) 53–57. https://doi.org/10.1016/j.talanta.2013.02.014.

[15]	V. Gómez-Jacinto, I. Garbayo, T. García-Barrera, C. Vílchez, J.L. Gómez-Ariza, Metallomic study of selenium biomolecules metabolized by the microalgae Chlorella sorkiniana in the biotechnological production of functional foods enriched in selenium, Pure Appl. 84 (2012) 269–280. https://doi.org/10.1351/pac-con-11-09-18.

[16]	V. Gómez-Jacinto, T. García-Barrera, I. Garbayo-Nores, C. Vilchez-Lobato, J.L. Gómez-Ariza, Metal-metabolomics of microalga Chlorella sorokiniana growing in selenium- and iodine-enriched media, Chem. Pap. 66 (2012) 821–828. https://doi.org/10.2478/s11696-012-0186-7.

[17]	D. Umysová, M. Vítová, I. Doušková, K. Bišová, M. Hlavová, M. Čížková, J. Machát, J. Doucha, V. Zachleder, Bioaccumulation and toxicity of selenium compounds in the green alga Scenedesmus quadricauda, BMC Plant Biol 9 (2009) 58. https://doi.org/10.1186/1471-2229-9-58.

[18]	T. Obata, H. Araie, Y. Shiraiwa, Bioconcentration mechanism of selenium by a coccolithophorid, Emiliania huxleyi, PCP 45 (2004) 1434–1441. https://doi.org/10.1093/pcp/pch164.

[19]	P.M. Neumann, M.P. De Souza, I.J. Pickering, N. Terry, Rapid microalgal metabolism of selenate to volatile dimethylselenide, Plant Cell Environ. 26 (2003) 897–905. https://doi.org/10.1046/j.1365-3040.2003.01022.x.

[20]	T.W.-M. Fan, S.J. Teh, D.E. Hinton, R.M. Higashi, Selenium biotransformations into proteinaceous forms by foodweb organisms of selenium-laden drainage waters in California, Aquat. Toxicol. 57 (2002) 65–84. https://doi.org/10.1016/S0166-445X(01)00261-2.

[21]	E.H. Larsen, M. Hansen, T. Fan, M. Vahl, Speciation of selenoamino acids, selenonium ions and inorganic selenium by ion exchange HPLC with mass spectrometric detection and its application to yeast and algae, J. Anal. At. Spectrom. 16 (2001) 1403–1408. https://doi.org/10.1039/b106355n.

[22]	T.W.M. Fan, A.N. Lane, R.M. Higashi, Selenium biotransformations by a euryhaline microalga isolated from a saline evaporation pond, Environ. Sci. Technol. 31 (1997) 569–576. https://doi.org/10.1021/es960471e.

[23]	N.R. Bottino, R.A. Zingaro, C.H. Banks, K.J. Irgolic, P. Micks, A.E. Wheeler, Selenium containing amino acids and proteins in marine algae, Phytochem. 23 (1984) 2445–2452. https://doi.org/10.1016/S0031-9422(00)84073-X.

[24]	J.J. Wrench, Selenium metabolism in the Marine phytoplankters Tetraselmis tetrathele and Dunaliella minuta, Mar. Biol. 49 (1978) 231–236. https://doi.org/10.1007/BF00391135.

[25]	C.M. Kayrouz, K.A. Ireland, V.Y. Ying, K.M. Davis, M.R. Seyedsayamdost, Discovery of the selenium-containing antioxidant ovoselenol derived from convergent evolution, Nat. Chem. (2024) 1–8. https://doi.org/10.1038/s41557-024-01600-2.

[26]	C.M. Kayrouz, J. Huang, N. Hauser, M.R. Seyedsayamdost, Biosynthesis of selenium-containing small molecules in diverse microorganisms, Nature 610 (2022) 199–204. https://doi.org/10.1038/s41586-022-05174-2.

[27]	B. Michalke, H. Witte, P. Schramel, Developments of a rugged method for selenium speciation, J. Anal. At. Spectrom. 16 (2001) 593–597. https://doi.org/10.1039/b100624j.

[28]	E. Vassileva, H. Dočekalová, H. Baeten, S. Vanhentenrijk, M. Hoenig, Revisitation of mineralization modes for arsenic and selenium determinations in environmental samples, Talanta 54 (2001) 187–196. https://doi.org/10.1016/S0039-9140(00)00652-4.

1. The identification of „Selenocysteic Acid“ was based on co-elution with cysteic acid (Wrench, 1978) and co-eluation with the compound formed by hydroperoxide oxidation of selenocystine (Wrench, 1978 and Bottino et al., 1984),
2. Identification of MeSeCys was based on changes in RT upon oxidation and comparison of these RT changes to what of S-methyl cysteine / S-methyl cysteine sulfoxide
3. Identification of MeSeMet was tentative and based on treating the sample with alkali and observing changes in RT to that of methionine, similar to what is observed for S-methyl methionine
